# The C-terminus of non-structural protein 1 (NS1) in H5N8 clade 2.3.4.4 avian influenza virus affects virus fitness in human cells and virulence in mice

**DOI:** 10.1080/22221751.2021.1971568

**Published:** 2021-09-05

**Authors:** Claudia Blaurock, Ulrike Blohm, Christine Luttermann, Julia Holzerland, David Scheibner, Alexander Schäfer, Allison Groseth, Thomas C. Mettenleiter, Elsayed M. Abdelwhab

**Affiliations:** a Institute of Molecular Virology and Cell Biology, Federal Research Institute for Animal HealthGreifswald-Insel Riems, Germany; b Institute of Immunology, Federal Research Institute for Animal HealthGreifswald-Insel Riems, Germany; c Friedrich-Loeffler-Institut, Federal Research Institute for Animal HealthGreifswald-Insel Riems, Germany

**Keywords:** Avian influenza virus, H5N8 clade 2.3.4.4, Interspecies Transmission, mammals, NS1, virulence, interferon antagonism, apoptosis

## Abstract

Avian influenza viruses (AIV) H5N8 clade 2.3.4.4 pose a public health threat but the viral factors relevant for its potential adaptation to mammals are largely unknown. The non-structural protein 1 (NS1) of influenza viruses is an essential interferon antagonist. It commonly consists of 230 amino acids, but variations in the disordered C-terminus resulted in truncation or extension of NS1 with a possible impact on virus fitness in mammals. Here, we analysed NS1 sequences from 1902 to 2020 representing human influenza viruses (hIAV) as well as AIV in birds, humans and other mammals and with an emphasis on the panzootic AIV subtype H5N8 clade 2.3.4.4A (H5N8-A) from 2013 to 2015 and clade 2.3.4.4B (H5N8-B) since 2016. We found a high degree of prevalence for short NS1 sequences among hIAV, zoonotic AIV and H5N8-B, while AIV and H5N8-A had longer NS1 sequences. We assessed the fitness of recombinant H5N8-A and H5N8-B viruses carrying NS1 proteins with different lengths in human cells and in mice. H5N8-B with a short NS1, similar to hIAV or AIV from a human or other mammal-origins, was more efficient at blocking apoptosis and interferon-induction without a significant impact on virus replication in human cells. In mice, shortening of the NS1 of H5N8-A increased virus virulence, while the extension of NS1 of H5N8-B reduced virus virulence and replication. Taken together, we have described the biological impact of variation in the NS1 C-terminus in hIAV and AIV and shown that this affects virus fitness *in vitro* and *in vivo*.

## Introduction

Avian influenza viruses (AIV) are members of the genus Influenza A Virus (IAV) in the RNA virus family *Orthomyxoviridae*. AIV infect a wide range of birds and mammals including humans. Wild birds are their natural reservoir, and infection is usually asymptomatic, with very few exceptions. In contrast, in poultry, AIV exhibit two pathotypes: low pathogenic (LP) and highly pathogenic (HP) [1, 2]. Interspecies transmission of AIV to immunologically naïve human populations can be devastating. As of 2019, there were 1096 fatalities out of 2644 laboratory-confirmed human infections with different AIV subtypes, resulting in a case fatality rate of 41.5% [3]. Similarly, AIV infections and associated mortality have been frequently reported in other mammals [4].

To date, 16 distinct hemagglutinin (HA) and 9 neuraminidase (NA) subtypes have been described for AIV. Each AIV carries one HA and one NA subtype, resulting in 144 possible HxNy combinations [5]. The genome of AIV is composed of eight gene segments, which together encode at least 11 viral proteins. Mutations in the HA receptor-binding domain and in the polymerase proteins are the major determinants for bird-to-human and bird-to-mammal transmission [6, 7]. However, studies have also shown that the non-structural protein 1 (NS1) plays an important role in the efficient replication and transmission of AIV in mammalian models without prior adaptation [8–13].

NS1 is expressed upon infection of host cells but is not incorporated into virions. However, one study also detected low amounts of NS1 protein within purified influenza virions [14]. NS1 consists of an RNA binding domain (RBD, residues 1–73) and an effector domain (ED, residues 88–230), which are connected by a linker (residues 74–87). NS1 is a multifunctional protein. The best-described of its functions is its ability to antagonize the interferon (IFN) response, either through binding of the RBD to RNA-sensors or interaction of the ED domain with different cellular proteins [15, 16]. This allows the replication of influenza viruses for almost 2 days before a sudden burst of immune responses, including the production of IFN, is triggered [17]. Although, NS1 typically has a length of 230 amino acids (aa), this is variable due to deletions in the linker region or changes in the positions of stop codons within the disordered C-terminal end (CTE) of the ED [18–20]. Sequence analysis showed that human influenza viruses from 1940 to 1980 possessed an NS1 of 237 aa (designated NS237), while viruses in the 1980s possessed an NS1 of 230 aa (designated NS230) [15]. Conversely, the pandemic H1N1 from 2009 has an NS1 of swine-origin that is 219 aa in length (NS219) due to an 11 aa deletion in the CTE (ΔCTE) [21]. Similarly, AIV of H5Nx exhibited 15 different NS1 lengths due to changes in the CTE [22]. The role of the CTE in the virulence of IAV, and particularly for AIV infection in mammals, is controversial. In mice, variations in the PDZ-recognition motif (aa 227–230) of NS1 contributed to the virulence of an LPAIV H7N1 [23] but it did not significantly affect the virulence of an HPAIV H5N1 [24]. Similarly, for an LPAIV H9N2, elongation of the NS1 from NS217 to NS230 or NS237 did not significantly affect virus replication in mammalian cells, nor did it affect virus replication or virulence in mice [25]. These paradoxical results might be explained by the use of different viruses in these experiments.

Since 1996/1997, H5Nx viruses of the Goose/Guangdong (Gs/Gd) lineage have continued to evolve in wild and domestic birds [26]. Recently, the H5Nx clade 2.3.4.4 has spread in global waves to wild and domestic birds from Asia into Europe, Africa and North America [27, 28]. Two 2.3.4.4 clades have been identified based on the genetic diversity of the HA gene: clade A in 2013–2015 and clade B in 2016–2020. In contrast to clade A viruses, H5Nx viruses of clade B have also been isolated from mammals including humans, pigs, mink, cats, and seals, indicating that they pose an increasing risk for mammalian species [29, 30](GISAID data). As a result, there are increasing calls for vigilance, including the assessment of the genetic changes that might increase the zoonotic potential of clade B 2.3.4.4 viruses [31]. In our recent study [22], we have shown that H5N8 clade A and B (designated hereafter as H5N8-A and H5N8-B, respectively) viruses have preferences for NS237 and NS217, respectively rather than the more common NS230. NS1 in both clades evolved quickly from NS230 and replaced their ancestors indicating selective advantages for virus replication. Indeed, we found that NS230 reduced virus virulence, replication, transmission and/or the efficiency of IFN antagonism in chickens and ducks. However, the impact of the variable CTE of H5N8 clade 2.3.4.4 viruses in mammals has not yet been studied. In this study, we (i) analysed the evolution of the NS1 CTE by analysing ∼51,000 NS1 sequences from 1902 to 2020, including those from human influenza viruses (hIAV), mammal-origin AIV and bird-origin AIV with an emphasis on the recent H5N8 viruses, (ii) studied the impact of the NS1 CTE on the expression of type I interferon (IFN) expression and the induction of apoptosis in human lung cells and (iii) assessed the biological impact of CTE variations on the virulence of different recombinant viruses in mice.

## Materials and methods

**Figure 1:**
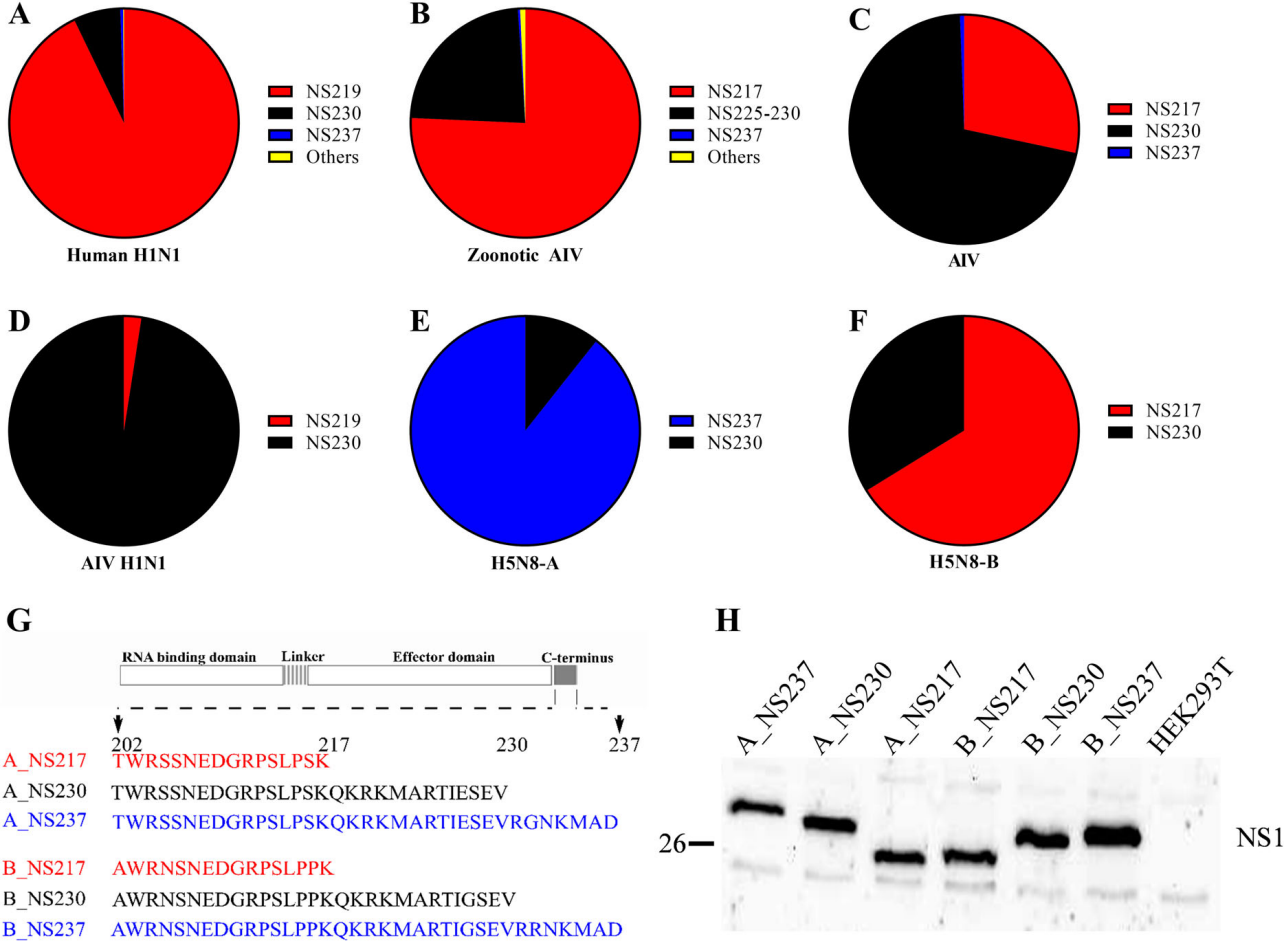
Sequence analysis of NS1 and expression of different H5N8 2.3.4.4 NS1 proteins in human cells. Sequences of full-length NS1 proteins of human H1N1 compared to AIV from birds, humans or other mammals from 1902 to 2020 were retrieved from GISAID on 28-03-2021. Sequences were cleaned from ambiguous residues and compared using Geneious version 11.1. The prevalences of NS1 of variable lengths in human H1N1 (A), zoonotic AIV (B), AIV (C), avian H1N1 (D) and H5N8 clade 2.3.4.4A (E) and 2.3.4.4B (F) viruses are shown. Schematic illustration of the C-terminus of recombinant viruses used in this study (G). NS1 expression in HEK293 T cells transfected with 1 µg of the indicated pCAGGS-NS1 plasmids for 24 h (H).

***Sequence analysis of NS1 sequences from influenza A virus of avian, human and mammalian origin.*** Sequences of full-length NS1 proteins from AIV in birds, humans or mammals, in addition to human H1N1 viruses, for the time-frame from 1902 to 2020 were retrieved from GISAID (retrieval date: 28-03-2021). Ambiguous and duplicate sequences were excluded and the remaining sequences were aligned using MAFFT [32] and analysed using Geneious version 11.1. The length of the NS1 and variations in the CTE were summarized (Figure 1, Supplementary Table S1). Maximum likelihood phylogenetic tree was generated using MEGAX for NS1 protein sequences of selected clade 2.3.4.4 H5Nx viruses of avian and mammalian origin. The tree was further edited using Inkscape (Supplementary Figure S1).

***Recombinant viruses and cells.*** A/turkey/Germany-MV/AR2487/2014 (H5N8) (hereafter designated A_NS237) and A/tufted duck/Germany/8444/2016 (H5N8) (hereafter designated B_NS217), belonging to clades 2.3.4.4A and 2.3.4.4B, respectively, were kindly provided by Timm C. Harder (National Reference Laboratory for Avian Influenza Virus, Friedrich-Loeffler-Institut (FLI)). Recombinant A_NS237 and B_NS217 viruses were previously generated [33]. The NS segments of these viruses were modified using site-directed mutagenesis to generate A_NS230 and A_NS217 from the A_NS237 plasmid, and similarly B_NS230 and B_NS237 were generated from B_NS217, by changing the stop codons in the CTE as described in a recent study [22] (Figure 1). All viruses were grown in 10-day old specific pathogen free (SPF) embryonated chicken eggs (ECE) according to standard protocols. Chicken fibroblast (DF1) cells, human adenocarcinomic alveolar basal epithelial (A549) cells, human embryonic kidney 293 T (HEK293 T) cells, Madin-Darby canine kidney (MDCK) cells and MDCK type II (MDCK-II) cells were provided by the cell culture collection in veterinary medicine of the FLI. Primary turkey embryo kidney (TEK) cells were prepared according to standard protocols, as previously published [34].

***Plaque assay.*** Standard plaque assays were used in this study to titrate the virus and assess cell-to-cell spread. Briefly, confluent MDCK-II cells were used for virus titration in 12-well plates. Ten-fold dilutions of the virus in phosphate-buffered saline (PBS) were incubated with the cells for 1 h at 37°C/5%CO_2_. Cells were washed twice with PBS after aspirating the virus dilutions and they were overlaid with semi-solid Bacto-Agar (BD, France) and minimal essential medium (MEM) containing 4% bovine serum albumin (BSA) (MP Biomedicals, USA). Cells were fixed after 3 days at 37°C, with 10% formaldehyde containing 0.1% crystal violet and kept at room temperature. 24 - 72 h later, the overlays were discarded and the diameters of 50 - 100 plaques were measured per virus to assess cell-to-cell spread. The Nikon NIS-Elements imaging software (Nikon, Germany) was used for this purpose. Plaque diameter is shown as the mean and standard deviation for each group.

***NS1 expression in human cells.*** To study the impact of the CTE on expression of NS1, A549 cells were transfected with different plasmids. Cloning of the six different NS genes (A_NS217, A_NS230, A_NS237, B_NS217, B_NS230, B_NS237) into pCAGGS was done after amplification of the NS genes from pHW-NS plasmids using specific primers containing NheI and SacI restriction sites (details available upon request). The pCAGGS-expression plasmid was kindly provided by Stefan Finke. The results of Sanger sequencing (Eurofins, Germany) for these constructs were evaluated in Geneious prime (2021.0.1), HEK293 T cells were transfected with 1 µg of each pCAGGS plasmid using Lipofectamine 2000 (Fischer Scientific, Germany) according to manufactures instructions. Transfected cells, as well as the supernatant, were harvested after 24 h at 37°C/5%CO_2_. Cells were centrifuged at 18000 xg for 5 min and were washed with PBS twice. Finally, pellets were resuspended in PBS and Laemmli-buffer (Serva, Germany) at a ratio of 1:1 and heated at 95°C for 5 min. Afterwards, sodium dodecyl sulfate polyacrylamide gel electrophoresis (SDS-PAGE) and Western blot were performed using a 12% polyacrylamide gel, as previously described [35]. Here, a primary polyclonal anti-NS1 antibody, which was kindly provided by Daniel Marc (Infectiologie et Santé Publique, French National Institute for Agricultural Research, France), was used at a dilution of 1:5000 in Tris-buffered saline with 0.1% Tween 20 (TBST) overnight at 4°C. A secondary peroxidase-conjugated rabbit IgG (Sigma, Germany) was used at a dilution of 1:20,000 for 30 min at room temperature. Detection was performed using Clarity Western ECL Substrate (BioRad, Germany).

***Replication kinetics in avian, mammalian and human cells.*** Primary TEK cells, DF1, MDCK, MDCK-II and A549 cells were inoculated with the different viruses using a multiplicity of infection (MOI) of 0.001 for 1 h at 37°C/5%CO_2_. After incubation, only TEK cells were treated with citrate buffer (pH 3.0) saline (CBS) for 2 min to inactivate extracellular virions. After washing the cells twice with PBS (for TEK cells after CBS treatment, for A549 cells after 1 h of infection), cells were covered with MEM containing 0.2% BSA. Infected cells were incubated for 24 h at 37°C/5% CO_2_. To block IFN-I signalling, A549 cells were pre-treated with 500 nM Ruxolitinib (Merck, Germany) (a JAK1/2 kinase inhibitor), or DMSO as a control, for 1 h in infection media with the indicated viruses at an MOI of 0.001. Treatment with Ruxolitinib or DMSO was also maintained at the same concentration following infection for the remainder of the experiment (i.e. 24 h). The experiment was performed in quadruplicates. Cells and supernatant were harvested and were stored at - 70°C until virus titre was determined using plaque assay.

***Detection of type I interferon responses in human lung cells.*** The relative expression of IFN-α and IFN-β mRNAs was measured in A549 and DF1 cells using generic RT-qPCR and/or luciferase reporter assay. For RT-qPCR, A549 and DF1 cells in 24-well plates were adjusted to 4 × 10^5^ cells per well and infected with the different viruses using an MOI of 0.1 for 24 h at 37°C/5%CO_2_ in triplicates. RNA extraction was done using TRIzol Reagent (Thermo Fisher Scientific, USA) and RNeasy Kit (Qiagen, Germany) according to the manufacturer’s instructions. The cDNA was transcribed from 400 ng of eluted RNA in a 20 µl reaction using the Prime Script 1st Strand cDNA Synthesis Kit (TaKaRA, Germany) with Oligo dT Primers. Quantification of IFN-α and IFN-β mRNA was performed using generic RT-qPCR with SensiFAST SYBR Lo-ROX Kit (Bioline, USA) or TaqMan® probes and previously published thermoprofiles [36, 37]. Relative expression of the IFN-mRNA in infected and non-infected cells was calculated using the 2^-(ΔΔct) method [38]. For luciferase reporter assays, A549 cells were transfected for 3 h before the medium was changed and the indicated viruses were added at an MOI of 0.1 for 20 h at 37°C. Luciferase reporter activity was measured as described below. Moreover, IFN-α and IFN-β protein expression were measured in the supernatant of infected A549 cells 24 h after infection with indicated viruses at an MOI of 0.1 using the LEGENDplex Human Anti-Virus Response Panel (BioLegend, Germany) according to the manufacturer’s recommendations.

***Detection of caspase-3 activity in human lung cells.*** A549 cells were infected with an MOI of 0.1 for 8 and 24 h at 37°C or treated with 20 µM (S)-(+)-Camptothecin (Sigma Aldrich, Germany) as a control. Cells and supernatants were harvested 8 and 24 h post infection (hpi), washed once with ice-cold PBS and lysed for 45 min on ice in cell extraction buffer (CEB, Invitrogen, Germany) supplemented with 1x cOmplete Protease Inhibitor Cocktail (Sigma-Aldrich, Germany) and 1 mM Phenylmethylsulfonylfluoride (Sigma-Aldrich). This lysis step was followed by a 10 min centrifugation step at 18,000 xg before mixing of the protein lysate with 4x SDS gel loading buffer (10% SDS (w/v), 40% glycerol (v/v), 20% β-mercaptoethanol, 0.008% Bromophenol Blue, 250 mM Tris-HCl pH 6.8). Samples were then heated at 95˚C for 5 min and proteins were separated in a 12% polyacrylamide gel by SDS-PAGE. Proteins were transferred onto a nitrocellulose membrane using a constant voltage of 15 V for 75 min (Bio-Rad) followed by a blocking step for 1 h at RT in 10% skim milk in TBST. Primary antibodies were incubated overnight at 4°C. Here, anti-NS1 (1:5,000); Vinculin (1:1000, Santa Cruz Biotechnology, Germany), NP (ATCC-HB65; 1:10,000) and caspase-3 (1:1,000 or 1:250, Cell-Signaling Technology, USA) primary antibodies were used. Secondary antibodies were incubated for 1 h at RT (anti-mouse (1:20000) and anti-rabbit (1:20,000 or 1:2,000), Cell-Signaling Technology, USA). Detection was performed with ECL-substrate using X-ray film (Fujifilm: Fuji Super RX-N 13 × 18 100 Bl).

***Flow cytometry.*** A549 cells were infected at an MOI of 0.1 with B_NS217, B_NS230 and B_NS237. 24 hpi, supernatants and cells were transferred into a collection tube and centrifuged at 3,000 xg for 5 min. Supernatant was discarded and cells were fixed with True-Nuclear-reagent (Biolegend, USA) for 30 min. Cells were washed with permeabilization buffer (Biolegend, USA) and stained with primary mouse-anti-NP antibody for 30 min followed by a secondary rat-anti-mouse IgG2a-Brilliant Violet 421 antibody (Biolegend) for 30 min. In a final staining step, the samples were incubated with an anti-active caspase-3 PE labelled antibody (Biolegend) for 30 min. After a final washing step with FACS-Buffer, cells were analysed using the LSRFortessa Flow Cytometer (BD). The percentage of active caspase positive cells was determined in both NP-positive (infected) and NP-negative (bystander) cell populations using the DIVA software package (BD).

***Luciferase assay.*** To determine whether the IFN-I (IFN-α and IFN-β) and/or NF-kB-induction pathway is inhibited by NS1, HEK293 T cells in 6-well plates were transfected with a plasmid DNA mixture containing 0.5 µg of a Firefly Luciferase (FFL) expressing reporter plasmids (i.e. p125:IFN-β-Pro-FFL, pIFNa-Pro-FFL, or p55:pNF-kB-Pro-FFL), 0.005 µg of pCMV-RL (normalization), 0.2 µg (pIRF-7) or 0.5 µg (pMDA5-delta, pTrif or pMyD88) as a trigger expression plasmid or 1 µg of poly(I:C) (InvivoGen, France), and 0.5 µg of pCAGGS plasmid containing one of the NS1 coding sequences (or empty vector as a control) using Lipofectamine 2000 transfection reagent. IFN-β promoter induction was analysed using p125:IFN-β-Pro-FFL as the reporter plasmid and poly(I:C), Trif or MDA-5 as a trigger. IFN-α promoter induction was analysed using pIFNα-Pro-FFL as the reporter plasmid and IRF7 as a trigger. NF-kB activation was analysed using p55 as the reporter plasmid and MyD88 or Trif as a trigger. 20 h post transfection cell extracts were prepared and Firefly and Renilla luciferase activities were measured using the Dual-Luciferase Reporter Assay System (Promega, Germany) according to manufacturer’s instructions. Luciferase activities were measured with a TriStar² LB 942 Modular Multimode Microplate Reader (Berthold, Germany). Firefly luciferase activity was normalized to Renilla luciferase activity. Induction of the promotor by a trigger was confirmed by comparing values for samples with and without transfection of the trigger plasmid. Promoter induction was set to 1 (100%) for the empty pCAGGS vector control with the given stimulus.

### Animal experiments

***Ethic statement.*** Animal experiments in this study were conducted following the German Regulations for Animal Welfare after obtaining the necessary approval from the authorized ethics committee of the State Office of Agriculture, Food Safety, and Fishery in Mecklenburg – Western Pomerania (LALLF M-V: permission number 7221.3-1-060/17) and approval of the commissioner for animal welfare at the FLI representing the Institutional Animal Care and Use Committee (IACUC). All animal experiments were carried out in the BSL3 animal facilities at the FLI.

***Experimental design.*** Five-week-old female BALB/C mice (Charles River, Germany) were allocated into groups of 3–5 mice per cage in closed ISOcage systems (Tecniplast, Italy). Mice were left to acclimatize for 5 days before infection and received food and water *ad-libitum*. The light and temperature were regulated automatically. On the day of infection (d0), the bodyweight (BW) of the mice was measured using a digital balance and they were numbered on the tail. Each mouse received either 10^3^ (*n* = 8) or 10^5^ (*n* = 5) pfu in 50 µL MEM containing antibiotics via the intranasal (IN) route. Sham mice (*n* = 8) were inoculated with 50 µL sterile MEM. All inoculation experiments were performed under mild anaesthesia using Isoflurane (CP-Pharma, Germany). BW gain, morbidity and mortality were recorded daily for 11 days post-inoculation (dpi). BW (in grams) relative to the BW at d0 was calculated for each mouse. Mice that lost more than 25% of the d0-BW value were humanely euthanized and scored as dead. At the end of the experiment, surviving mice were humanely euthanized using isoflurane and cervical dislocation. Mean time to death (MTD) was calculated for each group.

***Replication of H5N8 viruses in mice.*** To detect the amounts of viral RNA in the lungs, spleen and brains of mice, RT-qPCR targeting the M gene was performed as described [39] using SuperScript^TM^ III One-Step RT–PCR System with Platinum® Taq DNA Polymerase (Invitrogen, Germany) in an AriaMx real-time cycler as previously described [39]. Tissues were weighed and homogenized in 10% w/v of 1xPBS. Homogenates were centrifuged for 10 min at 15,000 xg and the viral RNA was extracted using the Viral RNA/DNA Isolation Kit (NucleoMagVET) (Macherey & Nagel, Germany). Standard curves were generated by testing serial dilutions of B_NS217 (10–100000 pfu) in each plate. To semi-quantify the viral RNA levels, the Ct-value were plotted and the corresponding dilution in the standard curve was automatically calculated. Results are shown as the mean and standard deviation of positive samples. Furthermore, infectious viral titres were determined in tissue homogenates using plaque assay in MDCK-II cells as described above.

***Seroconversion***. At the end of the experiment, blood samples were collected from the surviving mice after euthanization and decapitation. Serum samples were collected from whole blood at 4°C for 24 h followed by centrifugation. The commercially available “ID screen Influenza A Antibody Competition Multispecies kit” enzyme-linked immunosorbent assay (ELISA) (IDvet, France) was used to detect anti-AIV nucleoprotein using an Infinite® 200 PRO reader (Life Sciences, Tecan). According to the manufacturer’s recommendation 55% inhibition was set as the cut-off-point for positive samples, 45–55% was considered inconclusive and samples below 45% were considered negative.

***Statistics.*** Statistical analysis was performed with GraphPad Prism, Version 9.0.0. Plaque diameter as well as replication kinetics were analysed with a non-parametric Kruskal–Wallis (Dunn’s multiple comparison) and t-test. Fold-change in the IFN-induction and quantity of IFNs as well as caspase activity and viral load in different mouse organs were analysed using one-way ANOVA with post hoc Tukey’s test. Variation in the survival period of the mice was statistically analysed with the Log-rank (Mantel–Cox) test. Data were considered statistically significant at a *p* value < 0.05.

## Results

*In contrast to AIV of bird-origin, human H1N1, mammal-origin AIV and recent H5N8-B viruses exhibit a high prevalence of short NS1 forms.* We analysed NS1 sequences of human seasonal and pandemic H1N1 influenza viruses from 1902 to 2020 (*n* = 30766) and compared them to sequences of AIV of bird-origin (*n* = 17574), avian H1N1 (*n* = 661), recent H5N8 clade 2.3.4.4 viruses (*n* = 1070) and mammal-origin AIV from humans (zoonotic AIV) (*n* = 1626) or other animals (*n* = 162) (Figure 1A–F, Supplementary Table S1). The results showed that NS1 in human H1N1 viruses had three major forms: NS219, NS230 and NS237, with prevalences of 93.0%, 6.6% and 0.4%, respectively. However, there has been a striking shift since 2009. Before the emergence of pandemic H1N1 in 2009, NS219 was less prevalent (1.9%) than either NS230 (88.9%) or NS237 (9.2%). After 2009, NS219 was more prevalent (97.6%) than either NS230 (2.4%) or NS237 (0.02%) (Supplementary Table S1). Conversely, we found that AIV of bird-origin have the highest prevalence of NS230 (70.1%) followed by NS217 (28%) and NS237 (0.6%). Similarly, avian H1N1 viruses have only two forms (NS219 and NS230) with a clear preference to NS230 (97.5%) over NS219 (2.5%) (Figure 1D, Supplementary Table S1). These results indicate that recent hIAV prefer a shorter NS1 (i.e. NS219) in contrast to AIV which prefer the prototypical NS230.

Surprisingly, we found that H5N8-A from 2013 to 2015 contained NS237 (89.3%) and NS230 (10.7%), while H5N8-B from 2016 to 2020 contained NS217 (66.2%) and NS230 (33.8%) (Figure 1E and F, Supplementary Table S1), which indicate the existence of unique clade-specific preferences. The sequence and phylogenetic analyses further indicate that seal and human H5N8-B viruses isolated in Poland and Russia in 2016 and 2020, respectively also contain NS217 (Supplementary Figure S1). To gain an insight into a possible association between the length of NS1 and bird-to-mammal transmission, we analysed 1788 AIV NS1 sequences detected in humans (*n* = 1626) and animals (*n* = 162) from 1956 to 2020 (Supplementary Table S1). Indeed, we found that the prevalence of a short NS1 (i.e. 217) was higher (75.7%) than for NS225-NS230 (23.2%) or NS237 (0.3%) (Figure 1, Supplementary Table S1). These results indicated that transmission of AIV from birds to mammals is associated with a high prevalence of short NS1. Recent H5N8 viruses have unique clade-specific preferences towards long (H5N8-A) or short (H5N8-B) NS1 forms with unknown biological functions in mammals.

***Generation of recombinant H5N8 clade 2.3.4.4 A and B viruses and expression of NS1.*** To assess the impact of variation in NS1 length on the replication efficiency of H5N8 clade 2.3.4.4 viruses in mammals, we used six recombinant H5N8 viruses expressing NS1 proteins of different lengths [22]: three H5N8 clade A viruses (designated A_NS217, A_NS230 and A_NS237) and three clade B viruses (designated B_NS217, B_NS230 and B_NS237) carrying NS1 variants of 217, 230 or 237 aa, respectively, due to changes in the stop codons in the CTE (Figure 1G). H5N8-A and H5N8-B viruses used in this study shared 86.6–96.2% and 90.7–99.0% nucleotide and amino acid identities, respectively (Supplementary Table S2) and their NS1 clustered with those of other contemporary Eurasian viruses (Supplementary Figure S2). Viruses were propagated in ECE and virus titres were determined by plaque assay. Full genome sequences of these recombinant viruses excluded any unwanted mutations. Expression of NS1 was detected in HEK293 T cells at 37°C after transfection with pCAGGS-expression vector containing the different NS1 proteins. As expected, the molecular mass of NS1 correlated with the lengths of NS217, NS230 and NS237 (Figure 1H).

**Figure 2:**
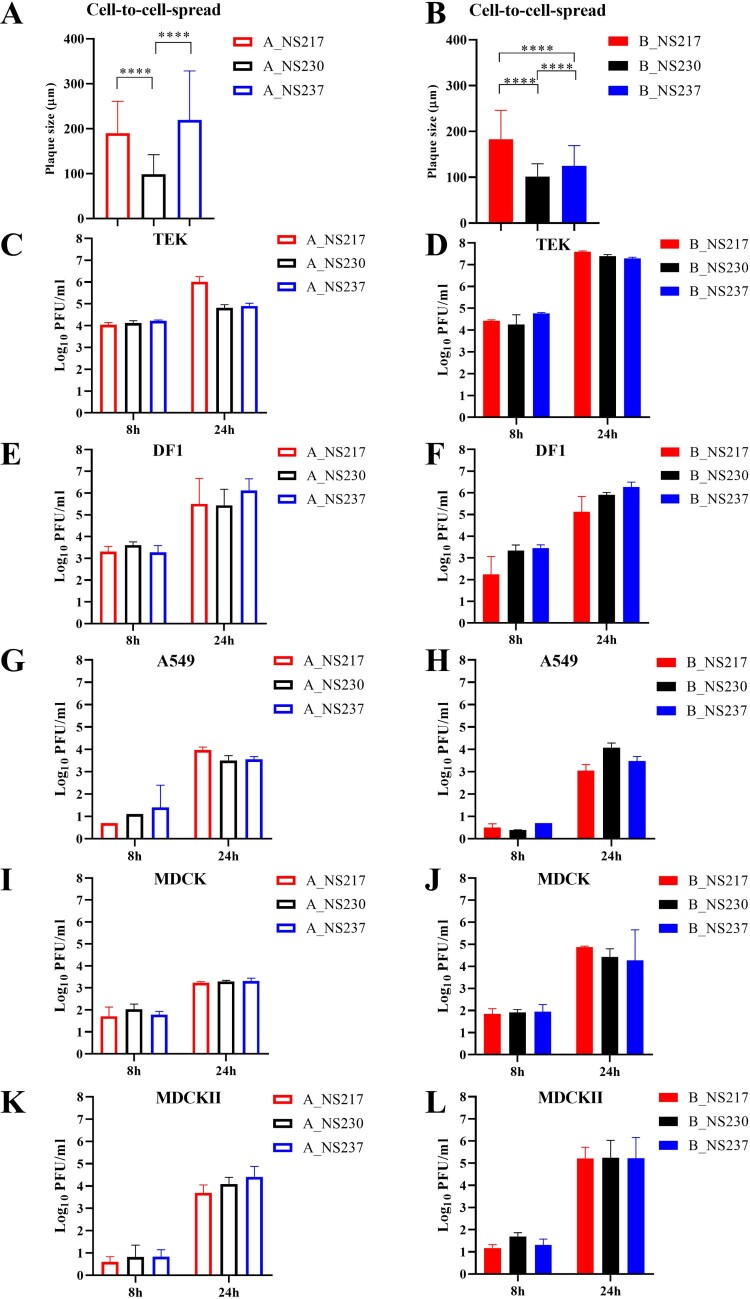
**The impact of NS1 C-terminus variations in cell-to-cell spread and replication of recombinant H5N8 viruses in cell culture.** Cell-to-cell spread was determined by measuring the diameter of 100 plaques after infection of MDCK-II cells with H5N8-A (A) or H5N8-B (B). Results are expressed as the mean and standard deviation (in µm). Replication kinetics in the indicated cell lines were performed at an MOI of 0.001 for 8 and 24 h. Virus titre was determined using plaque assay in MDCK-II cells. The assay was done in duplicates and repeated twice. Asterisks indicate statistical significance based on *p* values * < 0.05, ** < 0.01, ***  <  0.005, ****  <  0.0005. DF1= chicken fibroblast cell line, TEK = primary turkey embryo kidney cells, A549 = human lung adenocarcinoma cell line, MDCK = Madin-Darby canine kidney cell line, MDCK-II = MDCK type II (a derivative of the MDCK cell line).

***Changes in the CTE significantly reduced cell-to-cell spread without impacting virus replication in human cells.*** Alterations in the NS1 length of both H5N8-A and H5N8-B viruses reduced cell-to-cell spread as indicated by a smaller plaque size in MDCK-II cells (Figure 2A-B). The parental A_NS237 and B_NS217 viruses produced significantly larger plaques than their progeny expressing other NS1 variants (*p* < 0.0001). Viruses carrying NS230 produced the smallest plaques (*p* < 0.0001). We further compared the replication of these different viruses in avian (primary TEK and DF1), mammalian (MDCK and MDCK-II) and human A549 lung cells for 8 and 24 hpi. In TEK cells, the H5N8-A virus carrying NS217 replicated to significantly higher levels than other H5N8-A viruses at 24 hpi (*p* < 0.03) (*p* < 0.03) (Figure 2C–D). In DF1, A549, MDCK and MDCK-II cells, no significant differences were observed (Figure 2E–L). The replication of all viruses in human cells was significantly lower than in TEK cells. To test whether the poor replication in A549 was due to inefficient blocking of the IFN-I response, virus replication in Ruxolitinib-treated and untreated cells was compared. The presence of Ruxolitinib significantly increased the replication of A_NS230, A_NS217 and B_NS237 but had no significant impact on the replication of other viruses (Supplementary Figure S2). Taken together, NS230 significantly reduced cell-to-cell spread of both H5N8-A and H5N8-B, and extension of H5N8-B was disadvantageous for virus spread in mammalian cells with minimal impact on virus replication in human cells.

**Figure 3:**
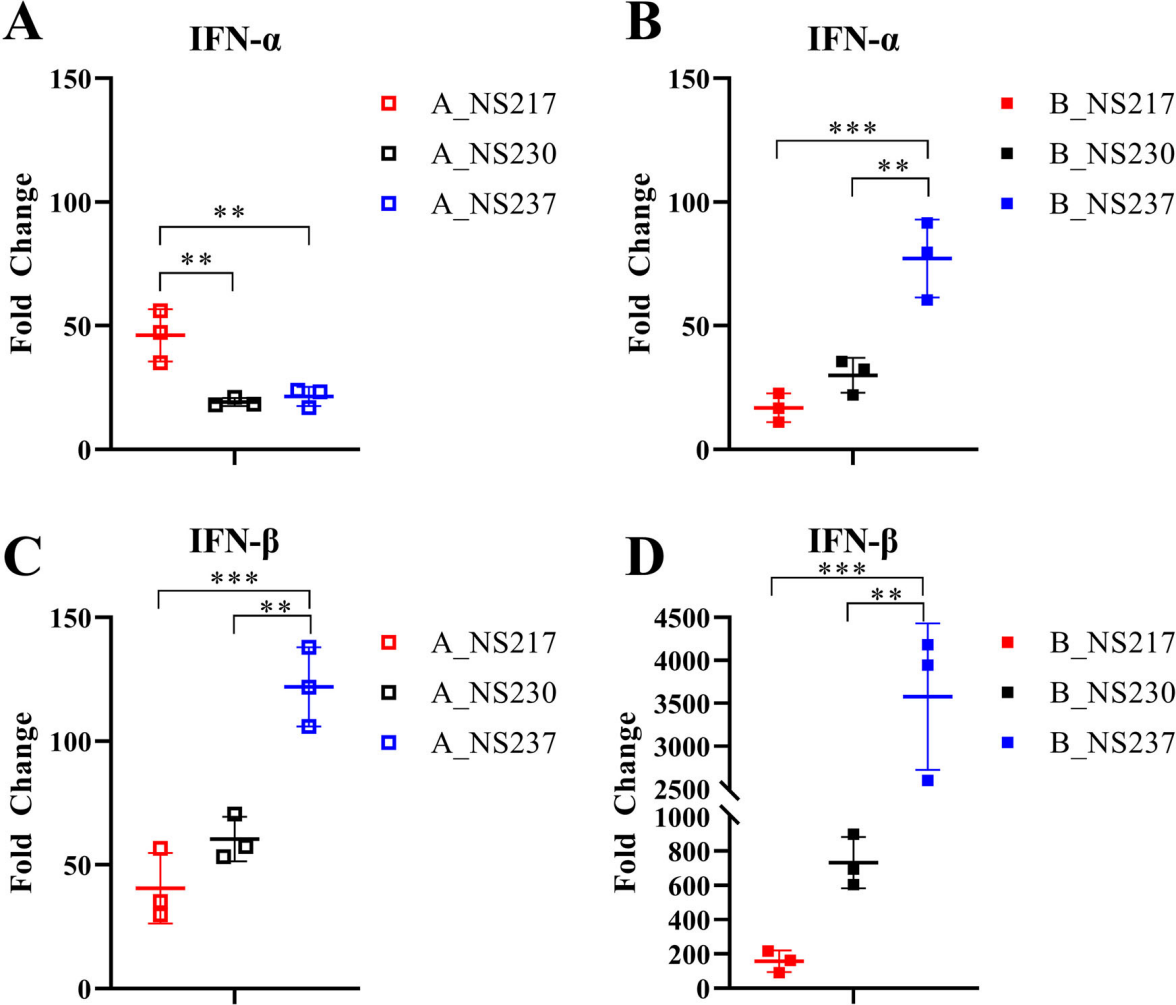
**The impact of NS1 C-terminus variations on interferon induction in human cells infected with H5N8 clade 2.3.4.4 viruses.** The relative expression of IFN-α (A, B) and IFN-β (C, D) mRNAs was measured in A549 cells infected with the different viruses using an MOI of 0.1 for 24 h. Relative expression of the IFN mRNAs in infected and non-infected cells was calculated using the 2^-(ΔΔct) method based on the results of three experiments. Asterisks indicate statistical significance based on *p* values *  <  0.05, ** <  0.01, *** < 0.005, **** < 0.0005.

***Extension of NS1 modulated the efficiency with which H5N8 viruses blocked IFN-α and IFN-β responses in human lung cells.*** It has been previously reported that the NS1 CTE impairs IFN-induction [40], however, other reports did not find any impact [24, 25, 41]. To test whether the H5N8 NS1 CTE can affect IFN-induction, we infected DF1 and A549 cells with H5N8-A and H5N8-B viruses carrying NS217, NS230 or NS237 and measured IFN-α and IFN-β mRNA levels using generic RT-qPCR (Supplementary Figure S3 and Figure 3A–D). In DF1 cells, shortening the H5N8-A NS1 significantly increased its ability to block IFN-β mRNA expression. Likewise, the extension of H5N8-B NS1 to 230 aa significantly reduced its ability to block both IFN-α and IFN-β mRNA expression (Supplementary Figure S3). In A549 cells, the results showed that viruses carrying NS217 (A_NS217 and B_NS217) were the most efficient at blocking the IFN-β response and that extension to NS230 and NS237 gradually reduced this efficiency (Figure 3C–D). A_NS237 was significantly more efficient than H5N8-A carrying shorter NS1 variants at blocking IFN-α response, while B_NS217 was more efficient at blocking IFN-α response than H5N8-B viruses carrying longer NS1 variants. To confirm these results, we also measured IFN-α and IFN-β promoter induction using luciferase reporter assays (Supplementary Figure S4) and measured the amount of IFN-α and IFN-β protein (Supplementary Figure S5) produced following infection of A549 cells with these viruses in two independent experiments. The results confirmed that the extension of H5N8-B NS1 to 230 or 237 aa reduced the efficiency with which it blocks the IFN response (Supplementary Figures S4 and S5). Together, these data show that extension of the NS1 CTE reduced the efficiency of H5N8-B viruses to block the induction of a type I IFN response in human lung cells. In contrast, shortening of NS1 of H5N8-A viruses reduced the virus’ ability to block the IFN-α response but enhanced its capacity to block IFN-β induction.

**Figure 4:**
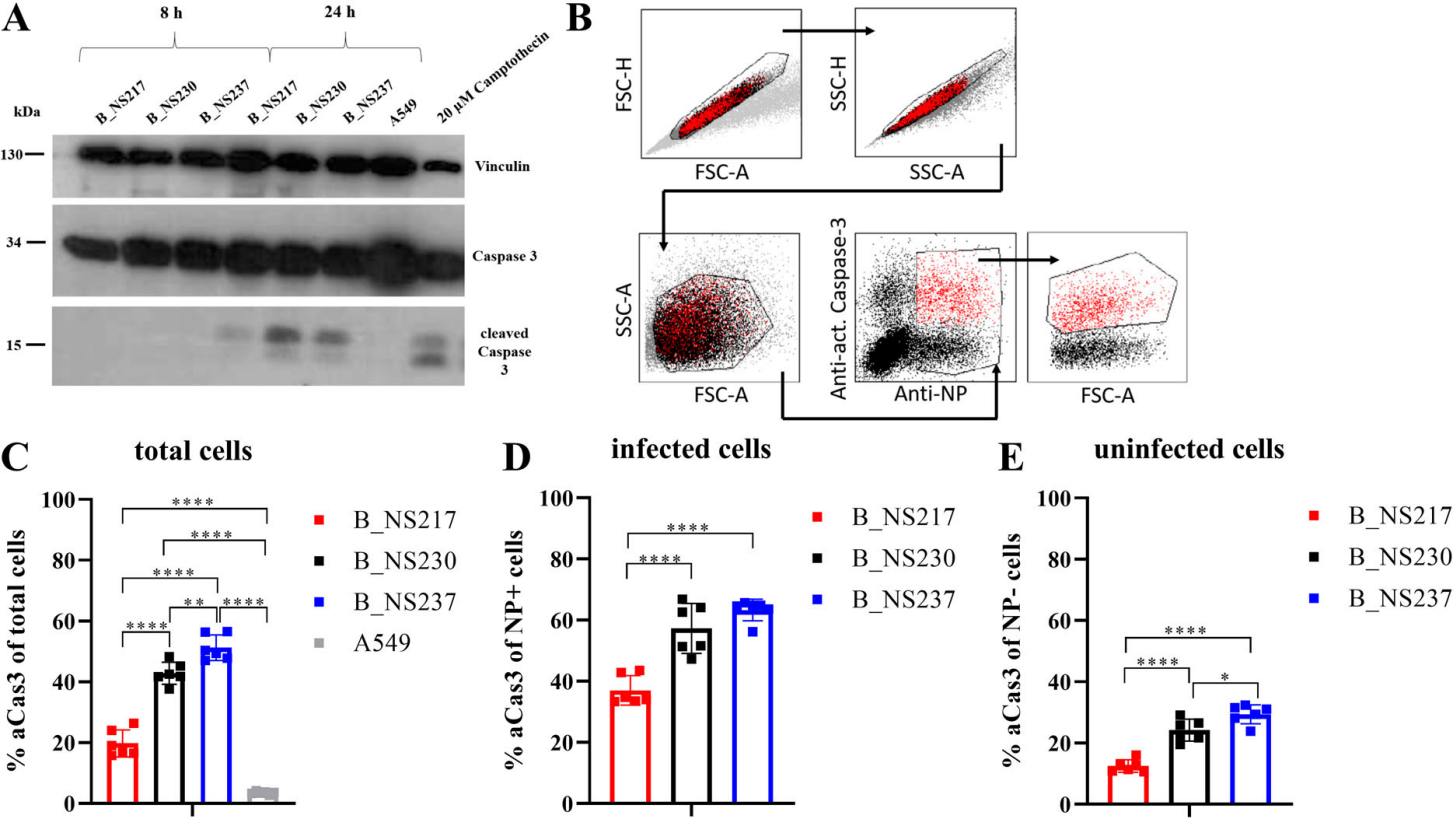
**The impact of NS1 C-terminus variation on apoptosis induction in human cells infected with H5N8 clade 2.3.4.4 viruses.** Active (i.e. cleaved) caspase-3 was detected in A549 cells after infection with an MOI of 0.1 for 8 and 24 h at 37°C. Cells treated with 20 µM Camptothecin were used as a positive control. Detection was performed with an anti-caspase-3 antibody using ECL-substrate and X-ray film (A). The amount of activated caspase-3 was quantified using flow cytometry after infection of A549 cells at an MOI of 0.1 for 24 h (B-E). Cells were stained with a primary mouse-anti-NP antibody (ATCC-HB65) and a caspase-3 PE labelled antibody. Signals were analysed via FACS with gating for either all cells (C), NP positive cells only (D), or NP negative cells only (E). Asterisks indicate statistical significance based on *p* values * < 0.05, ** < 0.01, *** < 0.005, **** < 0.0005.

***H5N8 viruses encoding shorter NS1 variants induced lower levels of active caspase-3 in human lung cells.*** It has been shown that H5N1 NS1 induces caspase-dependent apoptosis [42], and the PDZ domain in the CTE was associated with apoptosis induction [43]. To study the impact of variation in the NS1 CTE on apoptosis induction, we infected A549 cells for 8 and 24 h with B_NS217, B_NS230 or B_NS237 and detected caspase-3 using Western blot. No active (i.e. cleaved) caspase-3 (aCas3) was detected at 8 hpi, but all viruses induced aCas3 at 24 hpi. B_NS217 showed a weaker signal for aCas3 than either B_NS230 or B_NS237 (Figure 4A). To further quantify caspase 3 activation in both infected and non-infected (bystander) cells, we infected A549 cells with these different H5N8-B viruses and measured the levels of aCas3 and influenza NP using flow cytometry (Figure 4B–E). The results confirmed the WB findings that all viruses induced aCas3, but that the shorter the NS1, the less aCas3 was induced, regardless of the number of infected cells (Figure 4). These results indicate that H5N8-B viruses encoding a shorter NS1 variants induce apoptosis less extensively during infection, and that this is the case in both infected cells and in uninfected bystander cells.

**Figure 5:**
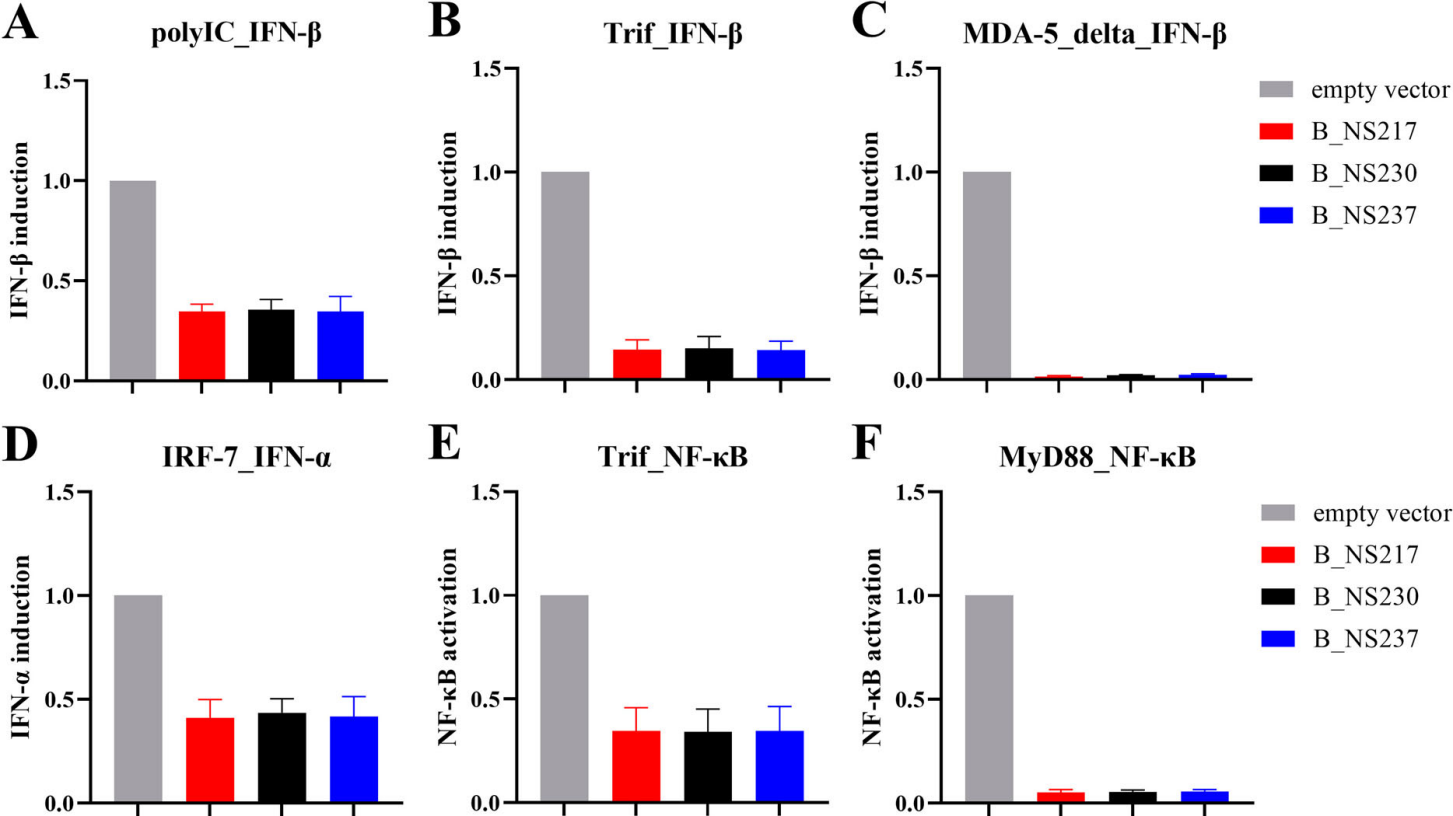
**Activation of IFN-I and NF-kB induction pathways in human cells after transfection with NS1 of H5N8 clade 2.3.4.4B.** To determine whether the IFN-I (i.e. IFN-α or IFN-β) or NF-kB-induction pathways are inhibited by NS1, HEK293 T cells in 6-well plates were transfected with a plasmid DNA mixture containing a reporter plasmid (i.e. p125:IFN-β-Pro-FFL, pIFNα-Pro-FFL or pNF-kB-Pro-FFL), pCMV-RL, a Trigger expression plasmid or poly(I:C), and a pCAGGS plasmid containing one of the NS1 coding sequences using Lipofectamine 2000 transfection reagent. Trigger molecules used to activate the different signaling cascades and the analysed promoters (i.e. reporter constructs) are indicated above the graphs (A-F). 20 h post transfection cell extracts were prepared and luciferase activities were measured using the Dual-Luciferase Reporter Assay System (Promega) according to manufacturer’s instructions. Firefly luciferase activity was normalized to Renilla luciferase activity. Promoter induction was set to 1 (100%) for the empty vector control with the given Trigger. Induction of the promotor by a trigger molecule was confirmed by comparing values for transfection of an empty pCAGGS vector with and without a trigger plasmid. FFL: Firefly luciferase; RL: Renilla luciferase.

***Changes in the NS1 CTE alone are not the main cause of differences in the inhibition of type I IFN-induction by NS1.*** Some studies have suggested that NS1 is unable to act directly as an inhibitor of apoptosis and IFN induction pathways which probably require the interplay or interaction of NS1 with other viral proteins (e.g. those encoded by the polymerase genes) [44, 45, 46, 47]. To study whether differences in the efficiency of H5N8 viruses encoding NS1 proteins of various lengths in regulating IFN- or apoptosis-induction was directly related to changes in the CTE, we transfected human cells with pCAGGS-NS (Figure 5A–F). We then measured IFN-α, IFN-β and NF-kB promotor activity using luciferase reporter assays and different triggers for induction of IFN-β (poly IC, Trif and MDA-5delta), IFN-α (IRF-7) or activation of NF-kB (Trif and MyD88). Surprisingly, no significant differences in the inhibition of IFN -induction or NF-kB activation were observed using the different NS plasmids (Figure 5), regardless of the trigger, the amount of NS plasmids or the cell line used (i.e. HEK293 T or A549) (data not shown). These results suggest that changes in the NS1 C-terminus are not the main factor affecting differences in the efficiency with which H5N8 viruses block type I IFN or NF-kB responses. Rather, differences in the interaction of the NS1 CTE with the products of other gene segments is likely the reason for this variable efficiency, although we also cannot exclude a role for effects on other signalling pathways (e.g. JAK-STAT).

**Figure 6:**
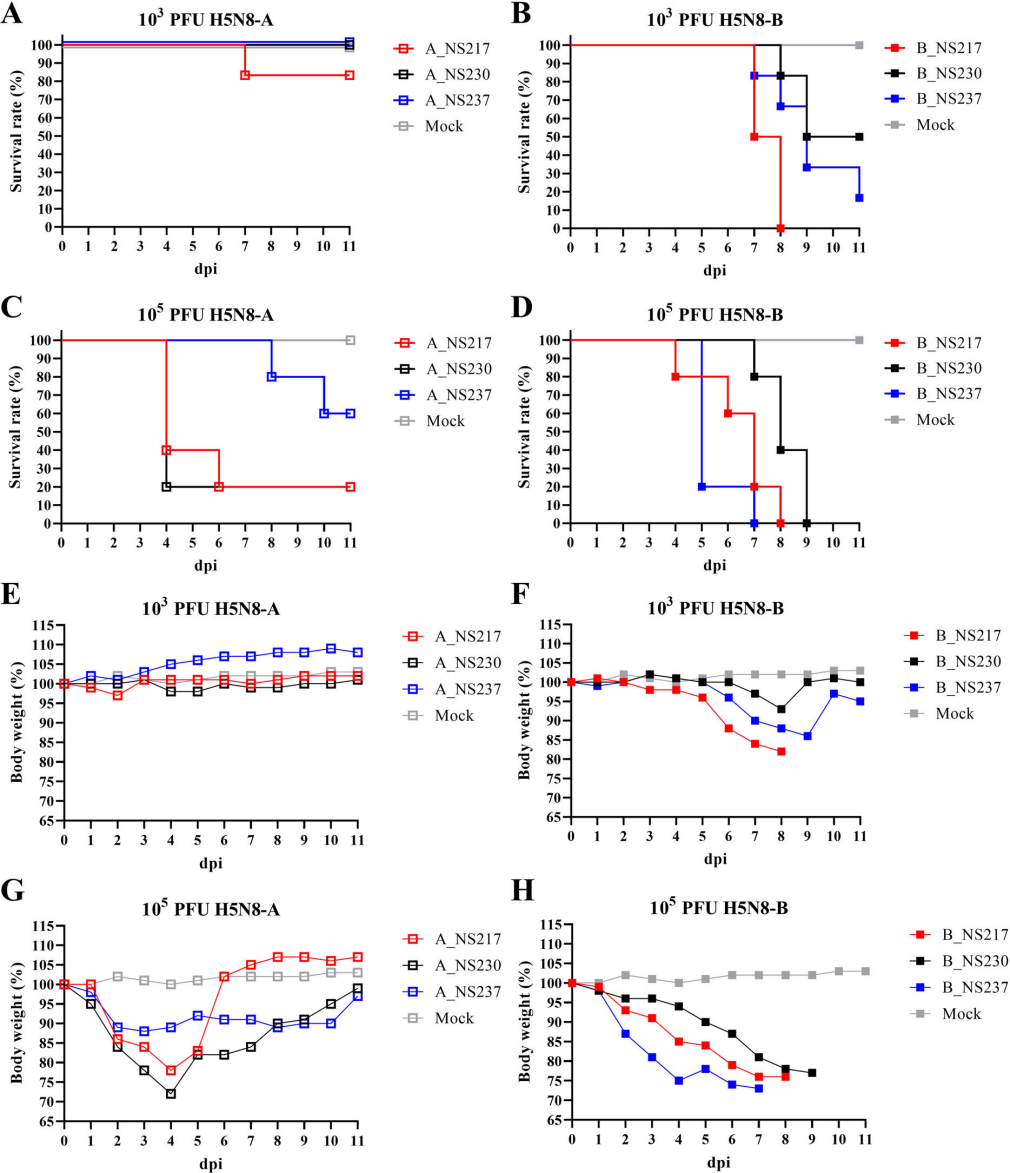
**The impact of NS1 C-terminus variations on survival time and bodyweight gain in mice inoculated with H5N8 viruses.** The estimated survival times are shown for BALB/C mice inoculated intranasally with a low-dose (103 pfu) (A, B) or a high-dose (105 pfu) (C, D) of H5N8-A (A, C) or H5N8-B (B, D). The relative bodyweight gain in BALB/C mice inoculated intranasally with a low-dose (E, F) or a high-dose (G, H) of H5N8-A (E, G) or H5N8-B (F, H) were calculated. A mock group were inoculated with 50 µL of sterile medium. Results show the relative mean bodyweight relative to the bodyweight immediately before infection (d0). Mice that lost more than 25% of their d0-bodyweight value were humanely euthanized and scored as dead. For clarity, standard deviations are not shown.

***H5N8 viruses carrying a short NS1 CTE caused higher mortality and reduced survival time in mice.*** To assess the pathogenicity and the impact of the NS1 CTE on virus virulence in mammals, mice were inoculated IN with low- and high-dose virus preparations. Clinical examination and assessment of survival were performed daily. After challenge with a low-dose, H5N8-A inoculated mice did not show clinical signs and survived until the end of the experiment, except for 1/5 mice inoculated with A_NS217 that died suddenly at 7 dpi (Figure 6A). Conversely, all B_NS217 inoculated mice died 7 and 8 dpi, with a MTD of 7.5 days after showing ruffled fur and mild to moderate depression (Figure 6B). Moreover, 2/5 and 4/5 mice inoculated with B_NS230 and B_NS237 died or had to be euthanized after showing neurological signs with MTD of 8.7 and 8.8, respectively. The survival time for mice inoculated with B_NS217 was significantly shorter than for mice inoculated with B_NS230 (*p* < 0.003) or B_NS237 (*p* < 0.02). After a high-dose challenge, 2/5, 4/5 and 4/5 mice died, with MTD of 9, 4 and 4.5 days, after inoculation with A_NS237, A_NS230 and A_NS217, respectively (Figure 6C). Mice in these groups did not show clinical signs but were humanely euthanized because they showed >25% BW loss. Conversely, all mice inoculated with B_NS217, B_NS230, and B_NS237 died, with MTD of 6.4, 8.2 and 5.4 d, respectively. The survival time for mice inoculated with B_NS217 or B_NS237 was significantly shorter than for mice inoculated with B_NS230 (*p* < 0.03) (Figure 6D). Mice in these groups showed severe depression and neurological signs. Taken together, H5N8-B was more virulent in mice than H5N8-A in mice. Further, H5N8 viruses carrying a short NS1 exhibited more severe clinical signs, a higher mortality rate and shorter survival times. The only exception was that the virulence of B_NS237 was comparable to that of B_NS217 after high-dose inoculation.

***H5N8 viruses carrying a short NS1 CTE caused greater bodyweight loss in mice in a dose-dependent manner.*** To assess the impact of variations of the NS1 CTE on the BW gain in mice inoculated with different viruses, mice were weighed daily. The changes in BW was calculated relative to day 0. Regardless of the infectious dose, mice inoculated with B_NS217 lost significantly more BW compared to A_NS237, and shortening the NS1 of the latter resulted in a more pronounced loss of BW. After inoculation with a low dose, H5N8-A inoculated mice did not exhibit a significant reduction in BW compared to the sham group, except for A_NS217 inoculated mice, which showed a transient reduction in BW at 1–2 dpi. Conversely, mice inoculated with B_NS217 showed the largest reduction in BW, followed by mice inoculated with B_NS237, and then B_NS230 (Figure 6F). After challenge with a high dose, mice inoculated with A_NS217 and A_NS230 lost more BW compared to A_NS237-inoculated mice, for which the reduction in BW gain was statistically significant at 3–5 dpi only (Figure 6G). Thereafter, surviving mice in these groups gained BW and recovered by 6 dpi (A_NS217) or 11 dpi (A_NS230 and NS237) (Figure 6G). Conversely, mice inoculated with B_NS217, B_NS230 or B_NS237 exhibited a drastic reduction in BW starting from 1 dpi until the day of death (Figure 6H). The largest reduction in BW was seen in mice inoculated with B_NS237 followed by mice inoculated with A_NS217, and then A_NS230 (Figure 6H). Taken together, H5N8-B inoculated mice exhibited a severe reduction in BW compared to H5N8-A inoculated mice after low-dose challenge. H5N8-A viruses carrying a short NS1 lost more BW compared to H5N8 viruses carrying NS237. Likewise, mice inoculated with H5N8-B carrying NS217 lost more BW compared to those inoculated with B_NS230. The impact of NS237 on BW loss after H5N8-B inoculation was dose-dependent.

**Figure 7:**
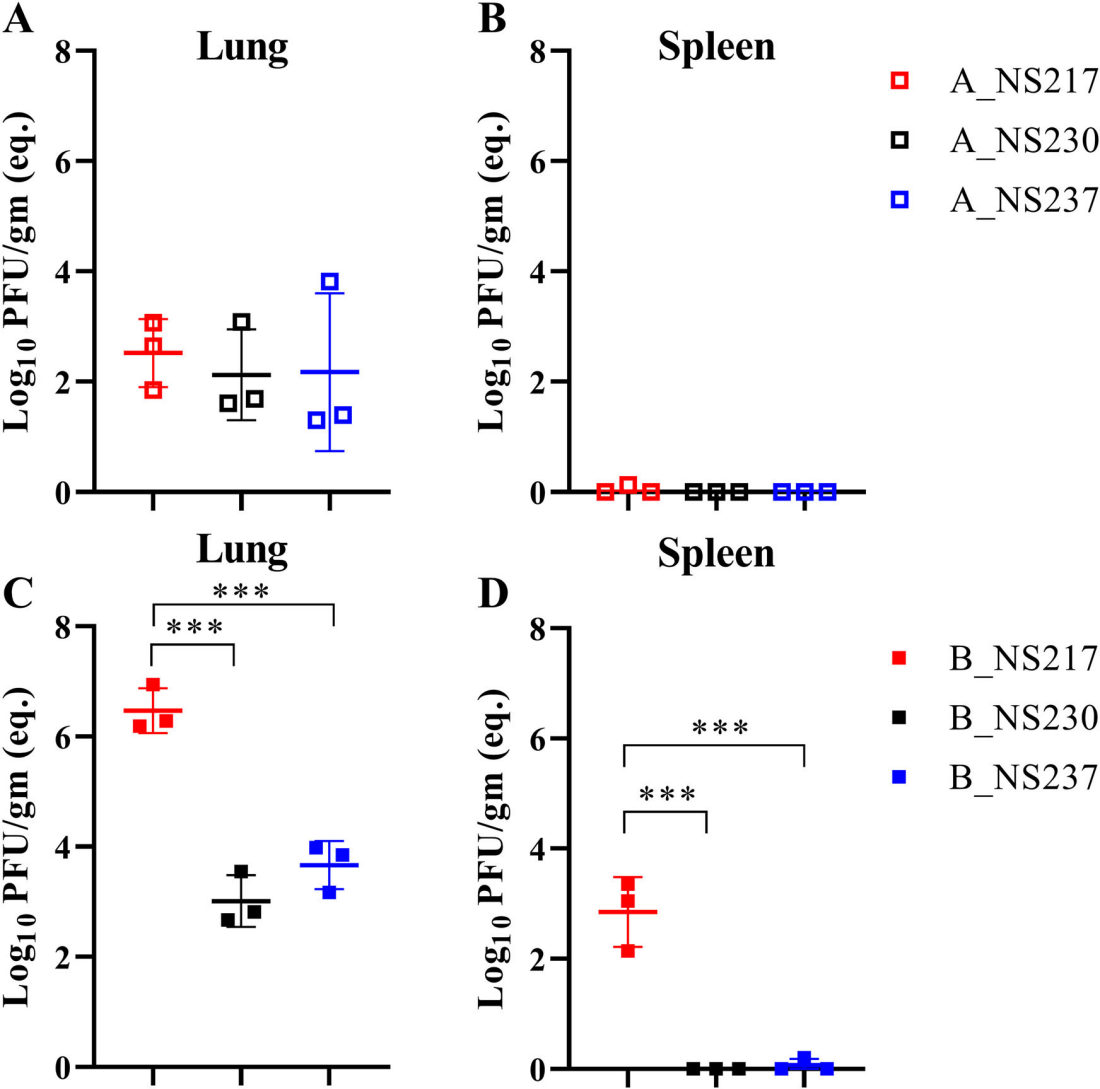
**The impact of NS1 on the replication of H5N8 viruses in mice.** Replication of the indicated viruses in mice inoculated intranasally with a low-dose was assessed in 3 mice per group sacrificed at 3 dpi (A-D). Viral RNA was extracted from the indicated organs and was detected using RT-qPCR with standard curves of serial dilutions of B_NS217 (10–100000 pfu) run in each plate. Results are shown as the mean and standard deviation of positive samples (A-D). Asterisks indicate statistical significance based on *p* values *** < 0.001.

*Clade B viruses replicated at higher levels than clade A viruses in mice, but elongation of the CTE reduced clade B virus replication.* To examine the replication of the different H5N8 viruses in lungs, spleen and brains without prior adaptation, groups of three mice were euthanized 3 dpi with a low-dose of virus and the viral RNA was quantified using RT-qPCR (Figure 7A–D) and viral load was measured by plaque test (Supplementary Figure S6). Using RT-qPCR, H5N8-A viruses were detected only in the lungs, indicating limited replication. In contrast, B_NS217 replicated to 10,000-fold higher titres than the H5N8-A virus (*p* < 0.005) (Figure 7A–B). However, extension of the NS1 of B_NS217 to NS230 or NS237 significantly reduced virus replication in the lungs by 1,000- and 500- fold, respectively (*p* < 0.005) and abolished virus replication in the spleen (Figure 7C–D). In the brain, viral RNA was detected in only 1/3 mice inoculated with B_NS217 (data not shown). Using a plaque test, infectious viral titres were detected in H5N8-B viruses only, with a similar pattern in titres to that obtained by RT-qPCR (Supplementary Figure S5). Antibodies were detected in all surviving mice, except in those inoculated with a low-dose of A_NS237 (*n *= 2/5), A_NS230 (4/5), or A_NS217 (3/5) (data not shown). Anti-NP antibodies were not detected in the sham group. Taken together, H5N8-B virus replicated to higher levels in the lungs and spleen compared to H5N8-A virus. Shortening the NS1 CTE did not have a significant impact on H5N8-A virus replication in mice, but extension of NS1 CTE reduced the replication of H5N8-B virus significantly.

## Discussion

AIV pose a serious public health threat either as a result of direct bird-to-human transmission or through the generation of pandemic influenza viruses after reassortment leading to the exchange of gene segments with hIAV. Because the human population is immunologically naïve to AIV, AIV infections may trigger an exacerbated immune response and can result in severe respiratory distress and death [48]. The continuous evolution of the panzootic H5N8 clade 2.3.4.4 viruses warrants vigilance to assess their potential zoonotic and pandemic risks [31]. Our sequence analysis revealed that the NS1 of H5N8-B viruses resembles that of human H1N1 and zoonotic AIV with a clear preference for a shorter NS1 protein. Conversely, AIV of bird-origin mostly possess a prototypical NS1 of 230-aa in length. Interestingly, compared to the vast majority of AIV, viruses of AIV H5N8-A and H5N8-B have evolved towards NS1 proteins with longer and shorter CTE, respectively, compared to the vast majority of AIV indicating biological advantages over NS230. We have recently shown that viruses carrying NS230 lost fitness in chickens and ducks [22]. However, the impact of these deletions (NS217) or extensions (NS237) in the CTE of AIV on virulence and replication of HPAIV in mammals is not adequately studied. Here, we did not find a significant impact on virus replication in human lung cells, but found that NS230 significantly reduced cell-to-cell spread in both H5N8-A and H5N8-B viruses. Similar to our results, Hale et al. [21] found that increasing the length of the 2009 H1N1 NS1 protein to 230 aa did not increase virus replication in human cells, while Jackson et al. [41] found that increasing the NS1 length of a laboratory WSN H1N1 increased the plaque size.

One of the well-described functions for NS1 is its ability to antagonize the interferon response, but the role of the CTE in the inhibition of IFN-induction is controversial. Mutations, deletions (6 or 10 aa) or extensions in the CTE of HPAIV H5N1, laboratory H1N1 or LPAIV H9N2 did not alter the IFN-β response in A549 cells [24, 25, 41]. Conversely, extension of the CTE in the pandemic H1N1-2009 increased the efficiency with which it blocked the IFN-β mRNA response in A549 cells [40]. Likewise, studies have shown that the NS1 of several AIV has pro-apoptotic activity [41], while the NS1 of other viruses mediates anti-apoptotic signalling responses [49, 50]. Here, we found that NS1 with a long CTE, regardless of the virus backbone, was significantly impaired in its ability to block IFN-β induction in human lung cells infected with H5N8 viruses. A similar pattern was obtained for apoptosis induction by H5N8-B viruses in both infected and non-infected bystander cells, whereby viruses encoding longer NS1 variants also induced higher levels of caspase 3 activation. These results may suggest that apoptosis induction is interferon-dependent or that both IFN-β and apoptosis are being regulated through a common pathway. IFN-dependent anti-apoptotic activity of a laboratory IAV (PR8/H1N1) after deletion in the NS1 has been previously reported [49].

Interestingly, in contrast to the positive correlation of IFN-β induction and the length of NS1, IFN-α induction was virus-dependent and not NS1 length-dependent, with increased IFN-α induction obtained after infection with H5N8-A carrying NS217 and H5N8-B carrying NS237. These results indicate that IFN-α and IFN-β are being antagonized by different mechanisms that are influenced differently by changes in the NS1 CTE, an observation that merits further investigation. The NS1 CTE contains residues, which interact with several host proteins including poly(A)-binding protein II (PABII), PDZ domain-containing proteins, importin-α and nucleolin [16]. Changes in the CTE could affect the interaction of NS1 with these proteins which might be compensated for by mutations in the NS1 or other viral proteins. Indeed, truncation of NS1 reduced the ability of H1N1-2009 to shut-off host mRNA transcription and trafficking, including for IFN mRNA. The ability of NS1 to block the IFN response was restored by binding to PABII after extension of the CTE [40] or by increased binding to the pre-mRNA processing protein CPSF30 via mutations in the ED [51, 52]. Two studies showed that deletion of 5 aa in the ED (residues 191-195) of swine-origin H5N1 virus attenuated its replication and virulence in mice and reduced its ability to block RIG-I mediated IFN-induction in mammalian cells [12, 13]. Moreover, recent studies have shown that interferon induction can be balanced or compensated for by other viral proteins (e.g. PB2, PB1-F2 or PA-X) after changes in the NS1 [47, 53]. This might explain the discrepancy between our results using infected and transfected cells to examine inhibition of the type I IFN and NF-kB pathways under our current experimental settings. It is worth mentioning that, independent of NS1, a contribution of the polymerase genes of H5N8 clade 2.3.4.4 (similar to our H5N8-B virus) to the induction of IFN-β has been recently reported [54].

Despite of the variations in IFN responses and caspase 3 induction, viruses in this study replicated at comparably low levels in A549 cells. Several studies have shown that some influenza viruses replicated at high levels in the presence of IFN, while other IAV were sensitive to IFN treatment and replicated poorly [55, 56]. Likewise, in one study the induction of apoptosis, mainly through activation of caspase 3, in A549 was found to be beneficial for replication of an HPAIV A/FPV/Bratislava/79 (H7N7) [57]. In Another study, the prevention of apoptosis conferred by NS1 was important for efficient influenza virus replication [50]. These results highlight the strain-dependent manner and specific mechanisms utilized by different influenza viruses to surmount the host responses. Here, and interestingly, the inhibition of IFN-I signalling in A549 by Ruxolitinib (a JAK1/2 kinase inhibitor) significantly increased the replication of A_NS230 (moderate IFN-β inducer), A_NS217 (strong IFN-α inducer) and B_NS237 (strong IFN-β inducer). Whether there is a correlation between NS1 CTE length and degree of activation of e.g. JAK/STAT pathway, remains to be investigated. Moreover, these results suggest that high levels of IFN probably reduced the replication of these three viruses in A549, however, it does not explain the low level of replication of H5N8-B despite the great ability to block IFN-induction. Beside the efficient interferon antagonism, the high replication of an AIV in human cells also requires modulating receptor binding affinity/avidity and polymerase activity which remain to be studied using H5N8 viruses in this study.

Compared to the low virulence and limited replication of H5N8-A in murine lungs, H5N8-B was more virulent in mice and viral RNA was detected in extrapulmonary tissues including the spleen and brain, indicating better replication in mammals without prior adaptation. This is in accordance with findings of previous studies that used the wild-type H5N8-B virus [58] or H5N8-A like viruses [59]. We also showed that H5N8-A and H5N8-B viruses with a short NS1 CTE (NS217) exhibited increased virulence in mice, as indicated by a high mortality rate, rapid onset of mortality and/or shorter survival times. Exceptionally, H5N8-B carrying NS237 was less virulent than H5N8 carrying NS217 after a low-dose inoculation but exhibited comparable pathogenicity after a high-dose inoculation. A possible explanation for this observation is variations in the balance between the immune system and virus replication. As seen from the experiment in human lung cells, H5N8-B carrying NS237 less efficiently blocked the induction of IFN and led to increased levels of apoptosis. Therefore, it is likely that mice were able to clear the virus after a low-dose inoculation, as seen by the reduced viral levels in the lungs, spleen and brains. However, a high-dose inoculation of mice with H5N8-B carrying NS237 probably triggered a “cytokine storm”, which caused the death of the mice in a similar fashion to what we observe for H5N8-B carrying NS217. This is consistent with the findings of other studies describing that deletion of 11-aa in the NS1 CTE increased pathogenicity of the pandemic H1N1-2009 virus in mice [40]. Conversely, in another study, a laboratory WSN strain with a truncated NS1 C-terminus (residues 227-230) showed reduced virulence and pathogenesis compared to viruses with a full-length NS1. Variation in pathogenicity was independent of the efficiency of IFN-antagonism by NS1 [41].

Clade 2.3.4.4 viruses were isolated from humans and other mammals [29, 30](GISAID data) and the hazard to the public health is not yet clear. Although, clade 2.3.4.4 viruses tested in this study did not replicate efficiently in human lung cells suggesting low zoonotic risk, however, H5N8-B virus was able to efficiently block the IFN induction in human cells and exhibited high virulence in mice. Therefore, the zoonotic potential of these viruses should not be totally neglected.

In summary, we found that H5N8-B clade 2.3.4.4 viruses have a preference for a short NS1, due to a truncation in the C-terminus, that is similar to human influenza viruses and zoonotic AIV. H5N8 viruses carrying a short NS1 exhibited a higher capacity for cell-to-cell spread, were more efficient at blocking the IFN-β response and showed reduced induction of apoptosis with minimal impact on virus replication in human lung cells. The truncation of NS1 also increased the virulence of H5N8 in mice, regardless of the virus backbone, and therefore it should be considered as a virulence marker for similar H5N8 viruses in mammals.

## Supplementary Material

R1_Suppl_Table_S2_clean_copy.docx

R1_Suppl_Table_S1_clean_copy.docx

R1_Suppl_Table_S2.docx

R1_Suppl_Table_S1.docx

Supplementary_legends.docx
