# Supplementary material for: The C-terminus of non-structural protein 1 (NS1) in H5N8 clade 2.3.4.4 avian influenza virus affects virus fitness in human cells and virulence in mice
Source: Emerg Microbes Infect. 2021 Sep 5;10(1):1760–76. doi: 10.1080/22221751.2021.1971568 (PMC8432360; doi:10.1080/22221751.2021.1971568)
Supplement: R1_Suppl_Table_S2_clean_copy.docx [file TEMI_A_1971568_SM4439.docx]

**C-Terminus of Non-Structural Protein 1 (NS1) in H5N8 Clade 2.3.4.4 Highly Pathogenic Avian Influenza Virus Affects Virus Fitness in Human Cells and Virulence in Mice**

**Claudia Blaurock^a^, Ulrike Blohm^b^, Christine Luttermann^b^, Julia Holzerland^a^, David Scheibner^a^, Alexander Schäfer^b^, Allison Groseth^a^, Thomas C. Mettenleiter^c^, and Elsayed M. Abdelwhab^a*^**

^a^Institute of Molecular Virology and Cell Biology, ^b^Institute of Immunology, ^c^Friedrich-Loeffler-Institut, Federal Research Institute for Animal Health, Südufer 10, 17493 Greifswald-Insel Riems, Germany

* Corresponding author: Elsayed M. Abdelwhab

Tel: +49 38351 7 1139;

Fax: +49 38351 7 1188

[sayed.abdel-whab@fli.de](mailto:sayed.abdel-whab@fli.de)

**Supplementary Table S2.** Identity matrices of H5N8-A and H5N8-B viruses used in this study

| Segment | Nucleotide identity (%) | Amino acid identity (%) | No of amino acid differences | Different amino acids*  (H5N8-A/H5N8-B residue number) |
| --- | --- | --- | --- | --- |
| PB2 | 86.6 | 97.4 | 20 | A/T106, I/V139, I/V260, T/V292, I/V338, R/K339, K/R340, Q/R508, L/Q522, I/T559, V/M575, I/V613, H/Q628, A/T666, D/F678, T/A684, R/K699, R/K702, V/A757, N/K759 |
| PB1 | 90.6 | 98.2 | 14 | K/T57, T/A110, I/V113, K/R168, K/R215, N/S216, V/I219, I/M317, A/T374, T/N375, E/D398, V/I667, N/S694, I/L753 |
| PA | 91.4 | 96.9 | 22 | T/A20, N/D27, K/E59, D/E101, K/R113, T/I129, I/T162, N/S184, K/E237, L/I242, M/L261, L/F290, P/L325, V/E327, V/A343, I/V354, P/S400, S/A404, S/A472, K/R615, S/G631, F/L672 |
| HA | 94.4 | 96.9 | 17 | K/E14, T/S94, D/N97, T/I114, A/T140, S/P141, P/S181, D/T195, V/I198, E/G268, I/V282, R/K325, V/I374, N/D475, K/Q490, G/E500, I/M531 |
| NP | 92.4 | 99.0 | 5 | N/D51, M/V105, V/F353, K/R452, S/N492 |
| NA | 96.2 | 97.0 | 14 | V/A8, T/M32, N/K46, V/I49, T/A81, S/A136, G/D263, R/Q264, T/M295, I/V303, T/A329, S/L397, Y/H450, T/M470 |
| M1 | 90.9 | 94.8 | 13 | I/V15, A/V33, K/R95, K/R101, R/K134, F/L144, M/I165, I/T168, S/A200, N/S207, N/S224, N/D232, N/K242 |
| M2 | 94.2 | 90.7 | 9 | T/N13, E/G14, R/K18, V/I28, N/S31, R/H45, I/V51, I/R61, E/G66 |
| NS1 | 92.8 | 90.8 | 20 | V/M6, S/L7, H/Y17, S/N48, D/G53, L/V65, G/E70, N/T80, I/V81, V/S84, T/S86, T/N127, I/T129, D/N139, T/A143, L/F166, I/N176, T/A202, S/N205, S/P216 |
| NEP/NS2 | 93.9 | 92.6 | 9 | V/M6, S/L7, L/M19, G/D27, E/G36, A/T48, N/S60, S/G70, M/V83 |

Identity matrices were calculated for the open reading frames and deduced amino acid sequences using Geneious.

* Positions of amino acid differences are from the first start codon of the main protein in each segment. Differences and numbering of residues in the HA are from the mature protein after removal of the signal peptide sequence.
