# Supplementary material for: The C-terminus of non-structural protein 1 (NS1) in H5N8 clade 2.3.4.4 avian influenza virus affects virus fitness in human cells and virulence in mice
Source: Emerg Microbes Infect. 2021 Sep 5;10(1):1760–76. doi: 10.1080/22221751.2021.1971568 (PMC8432360; doi:10.1080/22221751.2021.1971568)
Supplement: R1_Suppl_Table_S1_clean_copy.docx [file TEMI_A_1971568_SM4438.docx]

**C-Terminus of Non-Structural Protein 1 (NS1) in H5N8 Clade 2.3.4.4 Highly Pathogenic Avian Influenza Virus Affects Virus Fitness in Human Cells and Virulence in Mice**

**Claudia Blaurock^a^, Ulrike Blohm^b^, Christine Luttermann^b^, Julia Holzerland^a^, David Scheibner^a^, Alexander Schäfer^b^, Allison Groseth^a^, Thomas C. Mettenleiter^c^, and Elsayed M. Abdelwhab^a*^**

^a^Institute of Molecular Virology and Cell Biology, ^b^Institute of Immunology, ^c^Friedrich-Loeffler-Institut, Federal Research Institute for Animal Health, Südufer 10, 17493 Greifswald-Insel Riems, Germany

* Corresponding author: Elsayed M. Abdelwhab

Tel: +49 38351 7 1139;

Fax: +49 38351 7 1188

[sayed.abdel-whab@fli.de](mailto:sayed.abdel-whab@fli.de)

**Supplementary Table S1.** Prevalence of NS1 size of influenza A viruses from different hosts from 1902 to 2020

|  | **Host** | **NS1 amino acid length** | | | | | | Total |
| --- | --- | --- | --- | --- | --- | --- | --- | --- |
|  |  | **217** | **219** | **225** | **230** | **237** | Others |  |
| AIV | **H1** |  | 2 |  | 299 |  |  | 301 |
|  | **H2** | 1 |  | 1 | 556 |  | NS228 (1) | 559 |
|  | **H3** | 4 | 24 | 5 | 2218 |  | NS224 (1), NS228 (1) | 2253 |
|  | **H4** | 2 | 4 | 1 | 2140 | 6 | NS202 (1) | 2154 |
|  | **H6** | 920 |  | 15 | 1227 | 1 | NS202 (2), NS227 (1), NS228 (1) | 2167 |
|  | **H7** | 818 | 1 | 5 | 1625 | 2 | NS214 (1), NS216 (2), NS220 (24), NS224 (63), NS228 (1) | 2574 |
|  | **H8** |  |  |  | 194 |  |  | 194 |
|  | **H9** | 3091 | 12 | 53 | 917 | 93 | NS202 (1), NS212 (1), NS214 (2), NS220 (4), NS228 (5), NS229 (2), NS234 (2) | 4178 |
|  | **H10** | 73 |  |  | 1226 |  | NS207 (1) | 1300 |
|  | **H11** | 1 | 5 | 1 | 763 |  |  | 770 |
|  | **H12** |  |  | 1 | 340 |  |  | 341 |
|  | **H13** |  |  |  | 466 | 2 |  | 468 |
|  | **H14** |  |  |  | 42 |  |  | 42 |
|  | **H15** |  |  |  | 18 |  |  | 18 |
|  | **H16** |  | 3 |  | 252 |  |  | 255 |
|  | **Total** | **4910** | **51** | **82** | **12310** | **104** | **117** | **17574** |
|  | % | 28.0 | 0.3 | 0.5 | 70.1 | 0.6 | ≈ 0.7 | 100% |
| Human- Influenza | **Zoonotic AIV** | 1231 |  | 343 | 35 | 5 | NS202 (2), NS212 (1), NS215 (8), NS224 (1) | 1626 |
|  | **hH1N1 (1918-2008)** |  | 24 |  | 1145 | 119 |  | 1288 |
|  | **hH1N1 (2009)** |  | 4264 |  | 295 | 1 |  | 4560 |
|  | **hH1N1 (2010-2020)** |  | 24309 |  | 604 | 5 |  | 24918 |
|  | **Total** | **1231** | **28597** | **343** | **2079** | **130** | **12** | **32392** |
| Mammal-origin AIV | **bat** |  | 1 |  |  |  |  | 1 |
|  | **feline** | 2 |  | 16 | 6 |  | NS223 (1) | 25 |
|  | **dog** |  |  | 3 |  |  |  | 3 |
|  | **equine** | 1 |  |  | 17 |  |  | 18 |
|  | **ferret** |  |  | 6 | 4 |  |  | 10 |
|  | **meerkat** |  |  | 1 |  |  |  | 1 |
|  | **mink** | 9 | 1 | 8 | 4 | 1 |  | 23 |
|  | **rat** |  |  |  | 1 |  |  | 1 |
|  | **pika** | 1 |  | 5 |  |  |  | 6 |
|  | **stone martin** |  |  | 1 |  |  |  | 1 |
|  | **seal** | 1 |  |  | 6 |  |  | 7 |
|  | **swine** | 29 |  | 22 | 13 |  | NS220 (1), NS228 (1) | 66 |
|  | Total | 43 | 2 | 62 | 51 | 1 | 3 | 162 |
| AIV | **H1N1** |  | 16 |  | 645 |  |  | 661 |
|  | **H5N8-A** |  |  |  | 51 | 427 |  | 478 |
|  | **H5N8-B** |  | 392 |  | 200 |  |  | 592 |

Sequences were retrieved from GISAID and analyzed using Geneious. They represent all sequences in the GISAID to 28-03-2021.

NS225 and NS230 have a full CTE. Variation in length is due to deletion in the linker region
