# Supplementary material for: The C-terminus of non-structural protein 1 (NS1) in H5N8 clade 2.3.4.4 avian influenza virus affects virus fitness in human cells and virulence in mice
Source: Emerg Microbes Infect. 2021 Sep 5;10(1):1760–76. doi: 10.1080/22221751.2021.1971568 (PMC8432360; doi:10.1080/22221751.2021.1971568)
Supplement: Supplementary_legends.docx [file TEMI_A_1971568_SM4435.docx]

**Figure S1: phylogenetic tree of NS1 proteins of clade 2.3.4.4 H5Nx viruses**


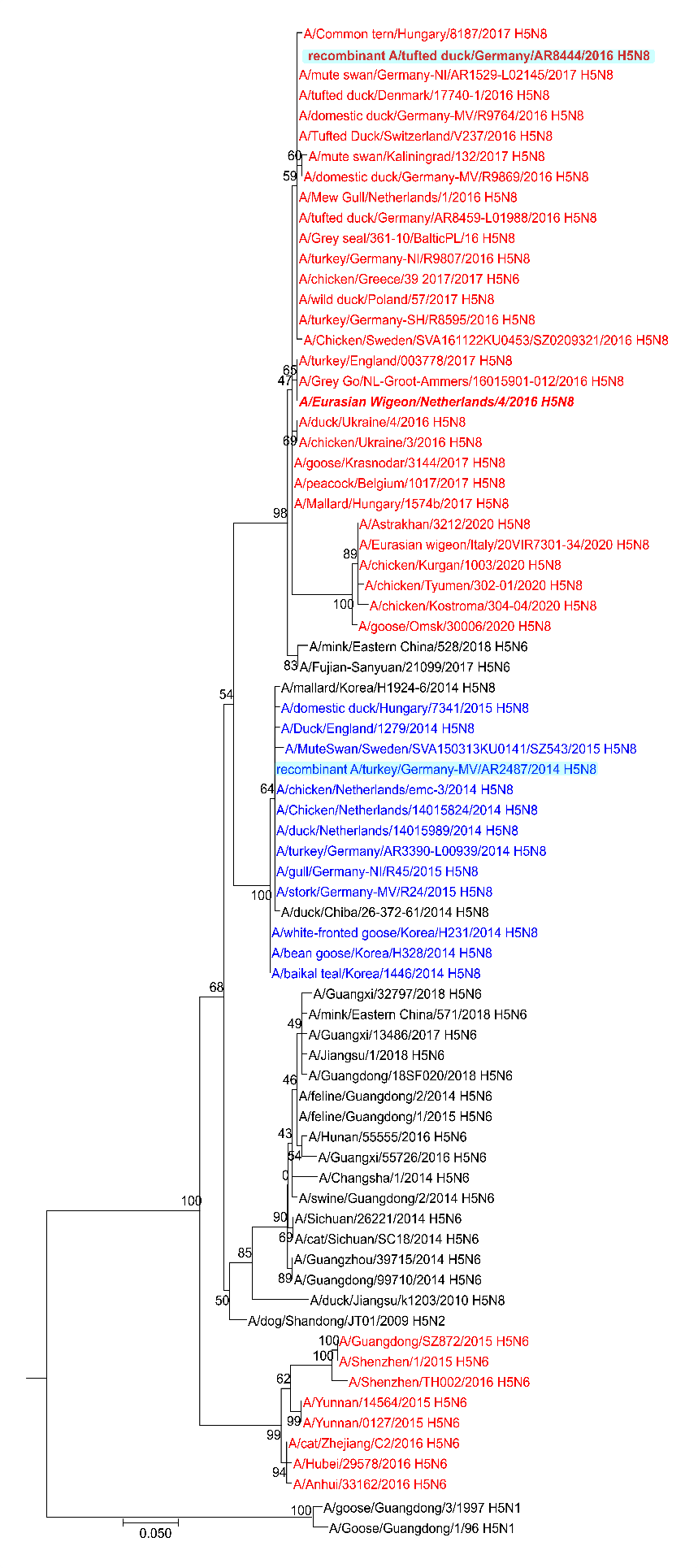


Maximum likelihood phylogenetic tree was generated using MEGAX software for NS1 protein sequences of selected clade 2.3.4.4 H5Nx viruses of avian and mammalian origin. The tree was further edited for publishing using Inkscape. Bootstrap values are shown on the branches. NS1 sequences of 217, 230 and 237 aa length are written in red, black and blue, respectively. The NS1 of H5N8-A and H5N8-B viruses used in this study are highlighted in cyan.

**Figure S2: the impact of Ruxolitinib on replication of H5N8 clade 2.3.4.4 in human cells**


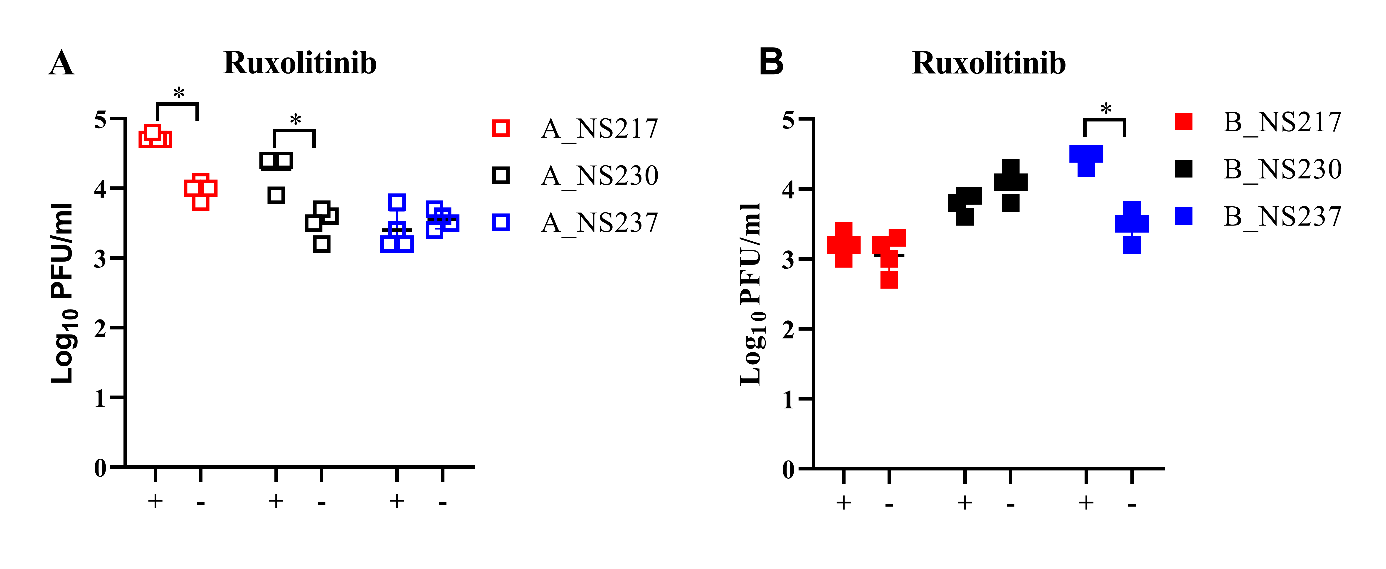


A549 cells were pre-treated with 500 nM Ruxolitinib or DMSO as a control, for 1 h in infection media with the indicated viruses at an MOI of 0.001. Treatment with Ruxolitinib or DMSO was also maintained at the same concentration following infection for the remainder of the experiment (i.e. 24 h). The experiment was performed in quadruplicates.

**Figure S3: The impact of NS1 C-terminus variations on interferon induction in chicken cells infected with H5N8 clade 2.3.4.4 viruses**


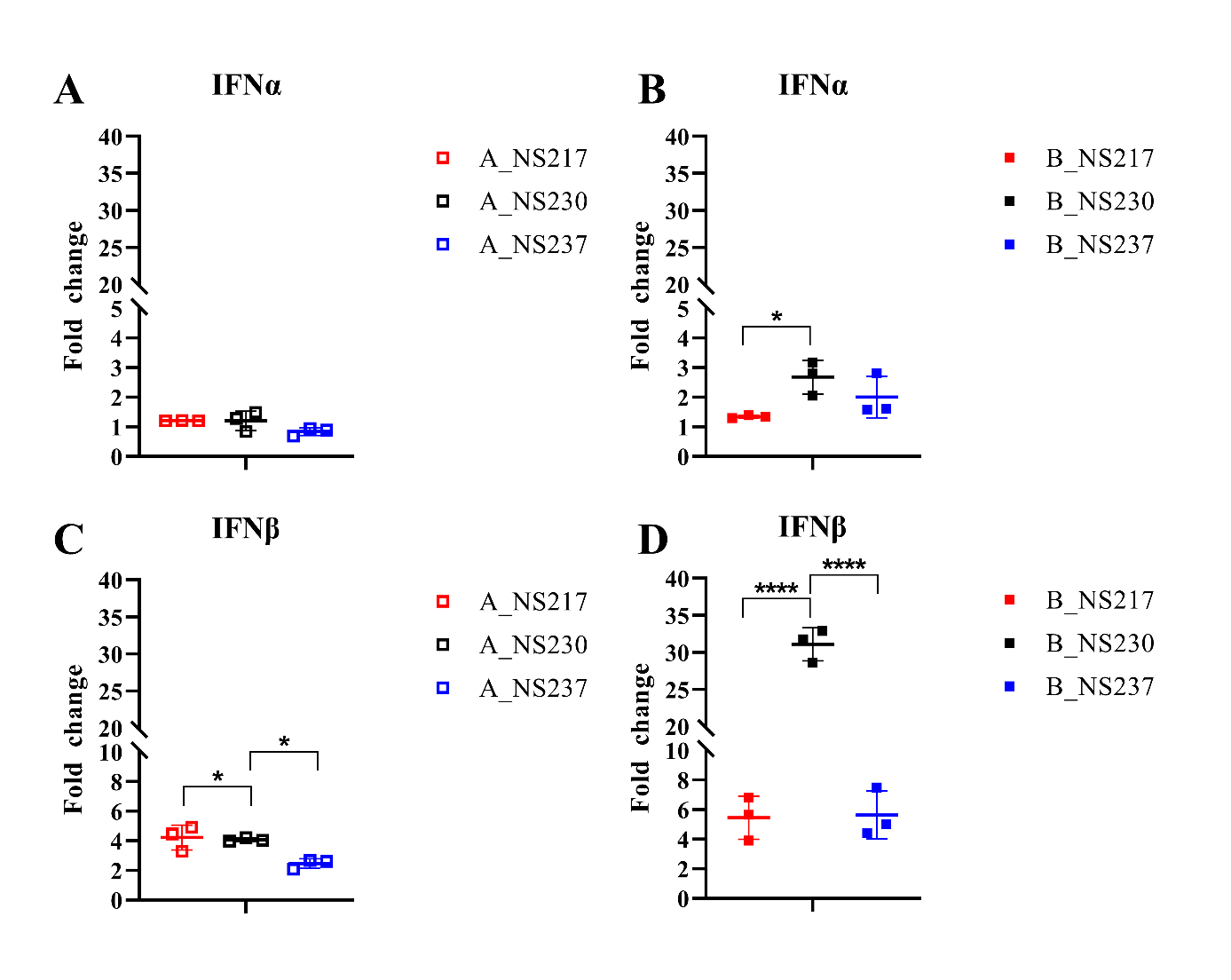
The relative expression of IFN-α (A, B) and IFN-β (C, D) mRNAs was measured in DF1 cells infected with the different viruses using an MOI of 0.1 for 24 h. Relative expression of the IFN mRNAs in infected and non-infected cells was calculated using the 2^-(∆∆ct) method based on the results of three experiments. Asterisks indicate statistical significance based on p values * < 0.05, **< 0.01, *** < 0.005, **** < 0.0005.

**Figure S4: Detection of type I IFN in human lung cells**


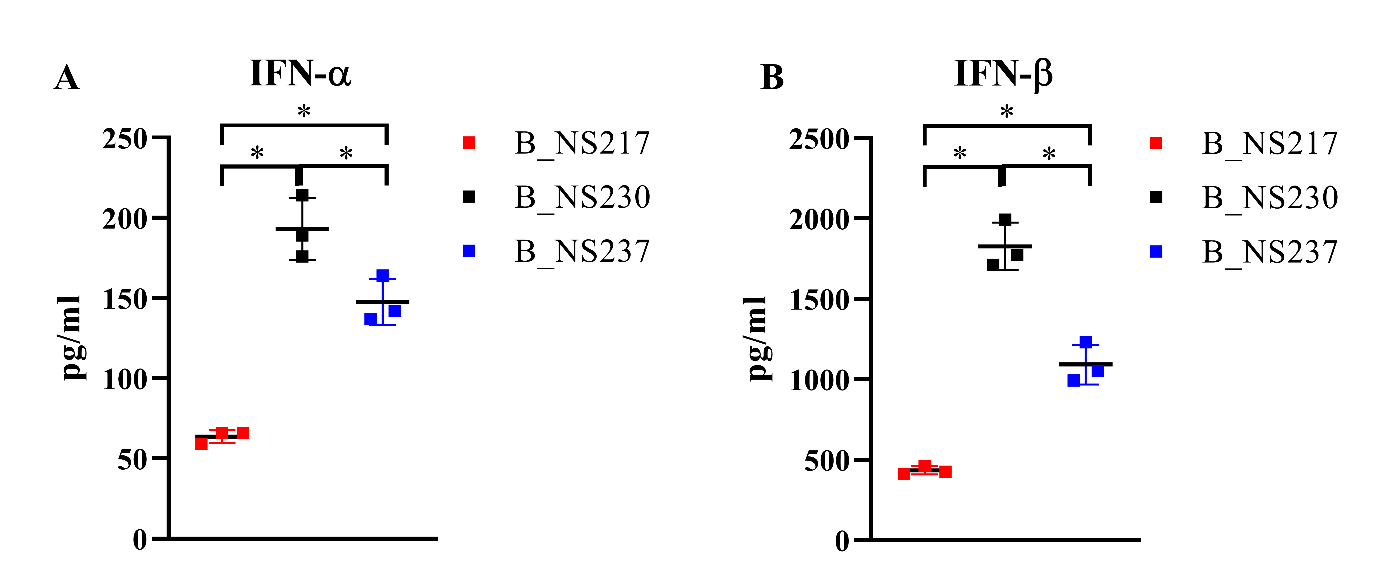


IFN-α and IFN-β protein expression were measured in the supernatant of infected A549 cells 24 h after infection with indicated viruses at an MOI of 0.1 using the LEGENDplex Human Anti-Virus Response Panel (BioLegend, Germany). The assay was run in triplicates. Asterisks indicate statistical significance based on p values * < 0.05, **< 0.01, *** < 0.005, **** < 0.0005.

**Figure S5: Detection of type I IFN-β activity using luciferase reporter assay**


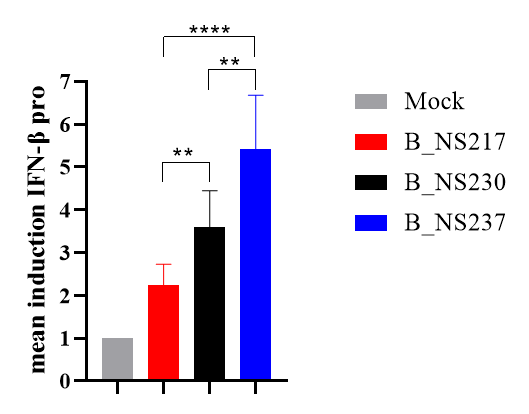


A549 cells were transfected for 3 h before the medium was changed and the indicated viruses were added at an MOI of 0.1 for 20 h at 37°C. Cell extracts were prepared and luciferase activities were measured using the Dual-Luciferase Reporter Assay System (Promega). Firefly luciferase activity was normalized to Renilla luciferase activity. Induction of the promotor by a trigger molecule was confirmed by comparing values for non-infected control cells.

**Figure S6: Virus titers in mouse organs after infection with clade 2.3.4.4 H5N8 viruses using plaque assay**


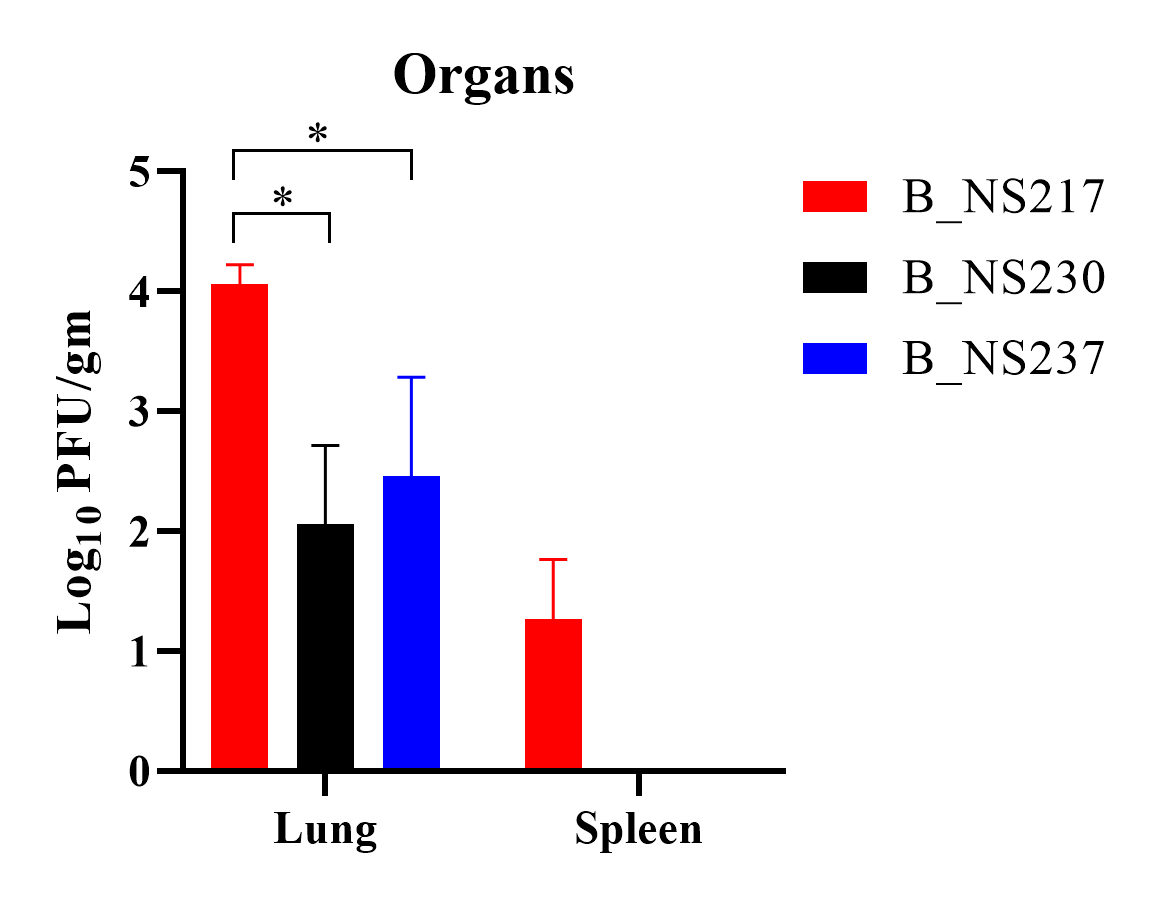


Replication of the indicated viruses in mice inoculated intranasally with a low-dose was assessed in 3 mice per group sacrificed at 3 dpi. Viral titers was determined from the indicated organs using plaque test in MDCK-II cells. Results are shown as the mean and standard deviation of positive samples. Asterisks indicate statistical significance based on p values * < 0.05.
